# NanoPrePro: a fully equipped, fast, and memory-efficient preprocessor for nanopore transcriptomic sequencing

**DOI:** 10.1093/bib/bbag063

**Published:** 2026-02-13

**Authors:** Chia-Chen Chu, Jhong-He Yu, Shang-Che Kuo, Fan-Wei Yang, Chia-Chang Lin, Chang-Hung Chen, Yi-Chen Wu, Cing Shih, Ying-Hsuan Sun, Te-Lun Mai, Ying-Lan Chen, Hsin-Hung Lin, Jung-Chen Su, Ying-Chung Jimmy Lin

**Affiliations:** Department of Life Science and Institute of Plant Biology, National Taiwan University, No. 1, Sec. 4, Roosevelt Rd., Daan Dist., Taipei 10617, Taiwan; Department of Pharmacy, National Yang Ming Chiao Tung University, No. 155, Sec. 2, Linong St. Beitou Dist., Taipei 11221, Taiwan; Department of Life Science and Institute of Plant Biology, National Taiwan University, No. 1, Sec. 4, Roosevelt Rd., Daan Dist., Taipei 10617, Taiwan; Department of Pharmacy, National Yang Ming Chiao Tung University, No. 155, Sec. 2, Linong St. Beitou Dist., Taipei 11221, Taiwan; Genome and Systems Biology Degree Program, Academia Sinica and National Taiwan University, No. 1, Sec. 4, Roosevelt Rd., Daan Dist., Taipei 10617, Taiwan; Department of Pharmacy, National Yang Ming Chiao Tung University, No. 155, Sec. 2, Linong St. Beitou Dist., Taipei 11221, Taiwan; Department of Life Science and Institute of Plant Biology, National Taiwan University, No. 1, Sec. 4, Roosevelt Rd., Daan Dist., Taipei 10617, Taiwan; Department of Life Science and Institute of Plant Biology, National Taiwan University, No. 1, Sec. 4, Roosevelt Rd., Daan Dist., Taipei 10617, Taiwan; Department of Life Science and Institute of Plant Biology, National Taiwan University, No. 1, Sec. 4, Roosevelt Rd., Daan Dist., Taipei 10617, Taiwan; Department of Life Science and Institute of Plant Biology, National Taiwan University, No. 1, Sec. 4, Roosevelt Rd., Daan Dist., Taipei 10617, Taiwan; Department of Forestry, National Chung Hsing University, 145 Xingda Rd., South Dist., Taichung 40227, Taiwan; Department of Life Science and Institute of Plant Biology, National Taiwan University, No. 1, Sec. 4, Roosevelt Rd., Daan Dist., Taipei 10617, Taiwan; Department of Biotechnology and Bioindustry Sciences, National Cheng Kung University, No. 1, University Rd., East Dist., Tainan 70101, Taiwan; Department of Agronomy, National Chung Hsing University, 145 Xingda Rd., South Dist., Taichung 40227, Taiwan; Department of Pharmacy, National Yang Ming Chiao Tung University, No. 155, Sec. 2, Linong St. Beitou Dist., Taipei 11221, Taiwan; Department of Life Science and Institute of Plant Biology, National Taiwan University, No. 1, Sec. 4, Roosevelt Rd., Daan Dist., Taipei 10617, Taiwan; Genome and Systems Biology Degree Program, Academia Sinica and National Taiwan University, No. 1, Sec. 4, Roosevelt Rd., Daan Dist., Taipei 10617, Taiwan

**Keywords:** Oxford nanopore technologies, transcriptome sequencing, preprocessing

## Abstract

NanoPrePro is a streamlined read preprocessor specifically designed for high precision in identifying full-length reads from Oxford Nanopore Technology (ONT) transcriptomic sequencing results, achieved through the precise identification of adapters/primers. However, the preprocessing of ONT reads has been a long-term neglected and ambiguous area without thorough and systematic investigation. Here, we developed NanoPrePro that outperformed the current best preprocessor, Pychopper, using simulated and real datasets. Through sequence similarity, adapter/primer location, and adapter/primer length, NanoPrePro exerted a self-optimizing function to extract the best parameters in each sequencing file for users to customize their analyses. Furthermore, NanoPrePro shows a 38-times faster speed with less memory cost. NanoPrePro can be regarded as the state-of-the-art preprocessor with forward adaptability of ONT sequencing.

## Introduction

Oxford Nanopore Technologies (ONT) cDNA long-read sequencing provides comprehensive structural information by sequencing each entire transcript, namely from 5′ end to 3′ end [1, 2]. Such technology ameliorates the problems of the subsequent transcriptomic analyses originally solely using short-read sequencing [3, 4]. Through the incorporation of the structural features from long-read transcriptome, numerous studies described the discovery of splicing variants [2, 5–7], transcript isoform quantification [2, 3], and other post-transcriptional events, including polyadenylation elongation [7, 8]. Along with this newly revealed knowledge on the transcripts, the structural annotations have been comprehensively updated in the organisms with available genome information [9–11], and *de novo* transcriptomic assembly can be carried out more precisely for the studies conducted in the organisms without genome sequences reported [12, 13].

**Figure 1:**
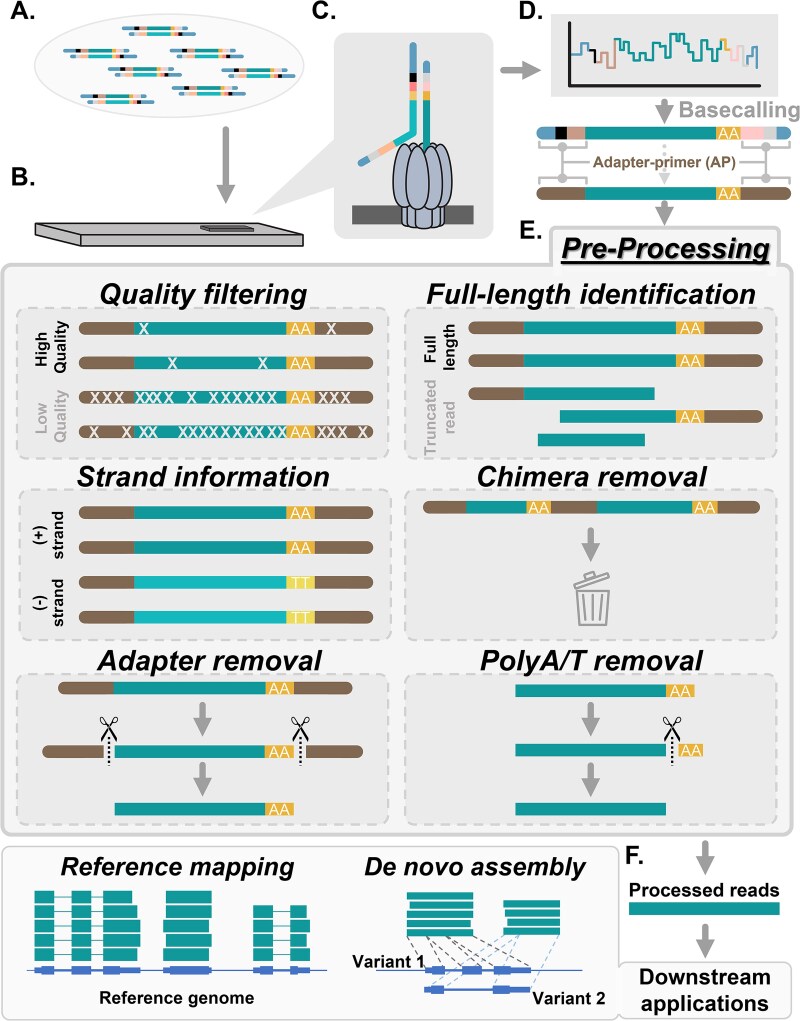
Workflow of ONT transcriptomic data analyses. The workflow for ONT transcriptomic data analyses was categorized into three parts: (A–D) ONT sequencing, (E) data preprocessing, and (F) downstream applications. The process began with constructing libraries (A), which were then loaded onto ONT flow cells (B). These libraries were passed through protein nanopores (C), generating electronic signals (D, top). These signals are subsequently decoded into nucleotide sequences through basecalling. (D, bottom) The sequencing results, often referred to as raw reads, consisted of the sequence of interest (turquoise), polyA/T sequences (yellow), and the flanking adapter/primer sequences (brown). (E) The six preprocessing tasks performed by NanoPrePro included: quality filtering (incorrect bases were represented by light-grey crosses), full-length read identification, strand information preservation, chimeric read removal, adapter removal, and polyA/T sequences removal. (F) The downstream applications of ONT transcriptomic data were classified into two main categories based on their requirement for a reference genome. Reference-based approaches involved mapping reads to a reference genome (left panel). Reference-free approaches interpreted data without genome information, such as *de novo* transcriptome assembly (right panel).

Library construction from the input RNA is required for ONT transcriptomic sequencing. Two major types of library construction kits are provided by ONT to meet different experimental requirements, including PCR-based kits to increase library yields from relatively limited amounts of RNA samples and PCR-free kits to avoid the bias and artifacts caused by PCR. Whichever sequencing kit is chosen, the constructed libraries are composed of cDNA (or RNA) flanked by the primers and adapters (Fig. 1A; see details in Fig. S1) and are subsequently loaded onto flow cells for sequencing (Fig. 1B). ONT sequencing represents the procedure as passing the cDNA/RNA library through the pores to generate electrical signals (Fig. 1C). Basecallers then decode the electrical signals into nucleotide sequences (Fig. 1D), as known as the raw reads, and also output quality scores of each nucleotide in each read to reflect the basecalling accuracy.

After basecalling, preprocessing of raw reads is crucial and necessary for downstream applications (Fig. 1E and F). Numerous studies described the necessity of different tasks to be conducted for preprocessing, and penalties were also reported if the tasks were neglected (Table S1) [3, 14–31]. However, each of these studies has not mentioned or integrated all the required tasks. We then summarized five necessary preprocessing tasks as follows (Fig. 1E).

First, quality filtering. An obvious drawback of ONT is its relatively high error rate on the raw reads, around 6%–14% [14, 32, 33]. Two reasons were found as the causes of the high error rates (mismatches, insertions, and deletions). First, the electrical signals are decoded into incorrect nucleic sequences during basecalling [14, 34], which can happen throughout the entire read. Second, the inconstant moving speed of nucleotides through nanopores leads to increased error rates on both read ends [14, 35]. Such unstable moving speed is due to the conformation differences between read ends (y shape) and read bodies (linear shape) (Fig. S1F) [35]. The reads containing many errors on the bases are regarded as low-quality reads, deteriorating the subsequent analyses. For example, low-quality reads tend to fail their alignment to the reference [3] and also cause the incapability of demultiplexing. Thus, quality filtering of ONT sequencing reads is a fundamental task to acquire high-quality reads for the robustness of downstream analyses (https://nanoporetech.com/community).

Second, full-length read identification. Although the ONT sequencing platform was developed to sequence ultra-long reads with theoretically unlimited read length, unexpected shorter reads were identified due to incomplete sequencing [15, 36, 37]. The truncated reads only contain the adapter/primer regions (or adapter regions) on the sequencing initiating ends, and the adapter/primer regions are absent on the sequencing terminating ends [16]. In other words, the sequencing of one read would be considered as completed only if the read possesses adapter/primer regions on both 5′ and 3′ ends, generally described as full-length reads. Using these truncated/non-full-length reads would cause problems, such as the misunderstanding of read-length distribution due to overestimation of the numbers of shorter reads [15], ambiguous assignment to the references during mapping [3], and misinterpretation of transcribed or coding regions in genome annotation [17, 38]. These reasons pointed out the necessity of identifying the adapter/primer region for full-length reads distinguishment.

Third, preservation of strand information. Genomic DNA is composed of two strands. The strands corresponding to translatable codes are defined as sense, and the complementary strands are antisense strands. The antisense transcripts transcribed from the antisense strand were widely identified in the species with complex genomes [19, 39]. In humans, antisense transcripts can be generated from more than 70% genome with high association with various diseases [20, 40, 41], and interfere with the functions of the sense transcripts through their degradation [19, 20]. Since antisense transcripts are transcribed from the overlapped genomic loci with sense transcripts, data misinterpretation in multiple situations may occur without the strand information. (i) The quantification of transcript abundance on both strands would be severely interfered [18, 42]. (ii) The sequences of the exon–intron boundaries from antisense transcripts would lead to the mischaracterization of the splice sites into the group of non-canonical splice sites (non-GUAG type) [43, 44]. (iii) The mis-annotation of splice variants on sense strands by the antisense transcripts. Thus, preserving the strand specificity of original transcripts is therefore considered critical for robust transcriptomic analyses.

Fourth, adapter/primer removal for clean reads. During the library construction, artificial nucleotide fragments, such as primers and adapters, are fused to the cDNA/RNA (Fig. S1), which can also generate electrical signals while passing through the nanopores (Fig. 1A–D). These artificial sequences incorporated into the raw reads have long been considered obstacles to high-quality transcript assembly and splice variant identification [27–29]. In contrast, clean reads derived from the raw reads with adapter/primer removed were reported to increase the subsequent mapping efficiency or *de novo* assembly quality of reads [30, 31].

Fifth, chimeric read identification. Each chimeric read contains two or more amplicons and has been reported to compose ~0.3%–8.1% of the ONT sequencing dataset [21, 23, 45–47]. The additional sets of adapters/primers, aside from both ends of reads, are the common features and are used for chimera identification [48]. These chimeras are a known issue caused during library construction and sequencing, and potentially hinder the subsequent assembly processes [21, 22]. The inclusion of chimeras would cause incorrect barcode assignment in demultiplexing [47] and lead to serious problems in quantitation-related applications in transcriptomes [23, 49]. Removal of the chimera was thus suggested by many previous studies to avoid bias as artificial recombinant molecules in downstream analyses [24–26].

Except for the five necessary tasks in preprocessing, many studies also remove the polyadenylated (polyA) tail (later as polyA) during preprocessing. In addition to the *in vivo* roles for the regulation of RNA stability and protein translation, polyA is also used for mRNA purification in most RNA sequencing kits. Since the polyA is not derived from the transcribed genome, its trimming has been incorporated as a default function for preprocessors of other sequencing platforms, such as Drop-seq (https://github.com/broadinstitute/Drop-seq), IsoSeq v3 (https://github.com/PacificBiosciences/IsoSeq), PRINSEQ [50], and Cutadapt [51]. It remains unknown whether polyA residual in ONT reads affects downstream analyses. The incorporation of polyA trimming as a required task during preprocessing remains an optional feature.

Six goal-oriented tools were developed to exert preprocessing for ONT reads. However, most of the tools only carry out single or a few functions for the five required tasks, leading to their incapability to extract properly processed reads (Table S2). Two of these seven tools, Prowler [52] and Filtlong (https://github.com/rrwick/Filtlong), filter the reads by quality scores. Besides basecalling, Guppy (https://nanoporetech.com/software/other/guppy) can also perform quality score filtering and adapter/primer removal, and these two functions can also be carried out by NanoFilt [53]. Dorado (https://github.com/nanoporetech/dorado), the latest basecaller, can further record the strand information, identify chimeric reads during the basecalling process. Porechop (https://github.com/rrwick/Porechop) conducts adapter/primer and chimeric read removal. In addition to the above-mentioned functions, Pychopper (https://github.com/epi2me-labs/pychopper), developed by ONT, can further recognize adapters/primers to extract full-length reads, while preserving the strand-specific information of each read. With the functions to execute all five required preprocessing tasks, Pychopper can be regarded as the current best preprocessor for ONT transcriptome data.

**Figure 2:**
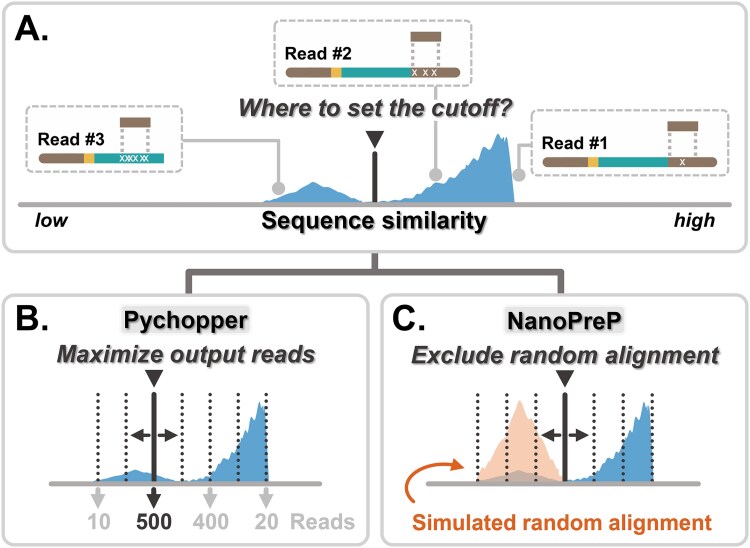
The key difference between the NanoPrePro and Pychopper algorithms. (A) The schematic diagram illustrated a common challenge in searching for adapters/primers on raw reads through alignment. For high-quality reads (example as Read #1), adapters/primers were identified with high sequence similarity to the theoretical sequences. For low-quality reads (example as Read #2), they were identified with lower sequence similarity. Truncated reads (example as Read #3) missed certain adapter/primer sequences, and random sequences were mistakenly identified as adapters/primers with much lower sequence similarity to the theoretical sequences. Sequence of interest, turquoise; polyA/T sequences, yellow; adapter/primer sequences, brown; incorrect bases, light-grey crosses. The major difficulty lay in setting an appropriate cutoff to differentiate real adapters/primers from random sequences. The downward-pointing triangle indicated the cutoff setting (black bold line). (B) Pychopper evaluated all possible cutoffs and selected the one that maximized the number of output reads. In this schematic example, 500 reads represented the maximum number of output reads. (C) NanoPrePro simulated random alignments and sought the optimal cutoff to exclude random alignments from real adapters/primers.

Besides quality filtering, the other five essential preprocessing steps can be achieved by identifying adapters/primers on the reads, which could be exerted by the accurate alignment of theoretical adapter/primer sequences to the reads (Fig. 2A). For ONT sequencing reads, however, this is particularly challenging because of their higher overall error rates [14–16] and the steep drop in sequence quality toward read termini [14, 18]. Sequencing errors, in the form of mismatches and indels, create inconsistencies between the theoretical and sequenced adapter/primer sequences, complicating the identification of genuine alignments. Among the six software, only Porechop and Pychopper provide adjustable parameters to carry out such functions. The adjustable parameters in Porechop were determined arbitrarily by users without sufficient characterization, which may not generate optimal outcomes. Pychopper is currently the only software that optimizes the parameters on adapter/primer identification based on a hypothesis that the optimal parameters could maximize the number of output “classified reads” (Fig. 2B) as a combination of full-length reads and rescued chimeric reads. Since ONT long-read sequencing has a high error rate, the key goal for accurate alignment of theoretical adapters/primers is to minimize false alignments. The hypothesis of the algorithm in Pychopper focuses on the maximization of output reads instead of the minimization of false alignment, which casts a shadow of doubt on the precision of their outcomes.

Here, we present NanoPrePro (https://github.com/Woodformation1136/NanoPrePro), a novel software equipped with functions to carry out all six key preprocessing steps, which implements a new algorithm for optimizing adapter/primer identification cutoffs using the alignment results from simulated true and false adapter/primer to find optimal parameters (Fig. 2C). Using both genome-based and reference-free applications of ONT transcriptomic data, we demonstrated that NanoPrePro outperforms Pychopper by providing significant improvements in data quality, especially in avoiding false adapter/primer alignment. In terms of computational resources, NanoPrePro runs more than 60 times faster than Pychopper with less memory consumption, which allows NanoPrePro to adapt to the growing throughput of ONT sequencing. Altogether, NanoPrePro is a fast and memory-efficient tool equipped with the necessary functions to obtain high-quality ONT transcriptomic data for accurate downstream analyses.

## Materials and methods

See Supplementary Methods.

## Results

### Algorithm design of NanoPrePro

The major algorithm of NanoPrePro was designed to handle the five preprocessing tasks besides quality filtering, which could be achieved by locating adapters and primers (later as APs) on the reads. APs are located by identifying the regions on the reads that show the highest similarity to the theoretical AP sequences through pairwise alignment. Pairwise alignment is well-suited for recognizing APs because it tolerates mismatches caused by sequencing errors. However, the problem with pairwise alignment is that it may recognize random sequences as APs if the actual AP is missing on a read (Read #3 in Fig. 2A). Thus, the challenge in identifying APs is to distinguish between alignment results for actual APs and random sequences.

**Figure 3:**
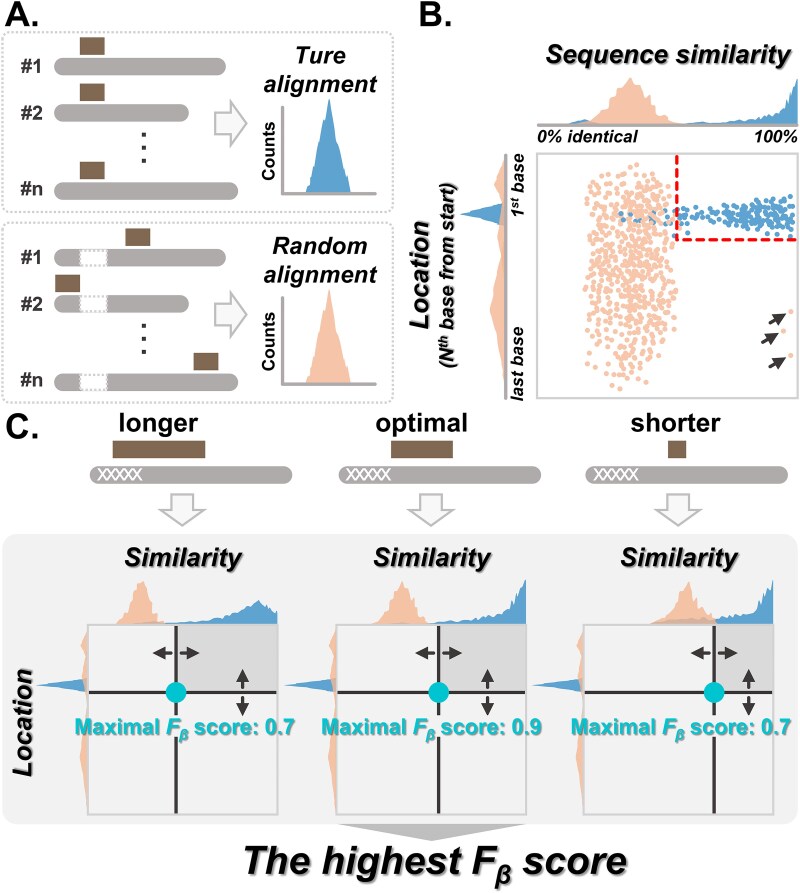
The coordination of three parameters in the NanoPrePro algorithm for optimizing adapter/primer identification. (A) The adapters/primers (brown) were aligned twice on each read (light-grey) to simulate true and random alignments. Edit distance was used as the scoring metric for alignment, and sequence similarity was calculated as 1 minus the edit distance divided by the adapter/primer length. The first alignments (top panel) were considered as true alignments, with the distributions (shown in blue) indicating sequence similarity or mapping locations. The second alignments (bottom panel) were performed after masking the region of the first alignment, and were regarded as random alignments, with the distributions shown in orange. The rectangles shown in dotted lines represented the masked region, corresponding to the aligned regions of the first alignment. (B) Schematic depiction of true and random alignment results within a two-dimensional representation, featuring adapters/primers sequence similarity and their locations. In the scatter plot, each dot represented an adapter/primer alignment result, with true alignments shown in blue dots and random alignments in orange dots. The x-axis illustrated sequence similarity in the alignment results, with identity levels increasing from left to right. The y-axis indicated their locations on the reads, ranging from the first base at the top to the last base at the bottom. The orange dots on the bottom right, highlighted by black arrows, represented rare instances of adapters/primers identified in the middle of the read with high sequence similarity. The histograms at the top and left of the scatter plot displayed the distributions of sequence similarity and locations, respectively. The red-dotted line indicated the optimal cutoffs for distinguishing between true and random alignments. (C) Schematic demonstration of how NanoPrePro determined adapter/primer length parameters, along with sequence similarity and location, to determine the final and optimal cutoffs. Different lengths of adapter/primer substrings (longer, optimal, and shorter as examples) were used to simulate true and random alignments. The F_β_ score served as the indicator for evaluating the performance of cutoffs. For different length of adapter/primer substrings, combined with sequence similarity and location parameters, a maximal F_β_ score was identified. For example, the maximal F_β_ scores for both longer and shorter adapter/primer substrings were 0.7, while the optimal adapter/primer substrings achieved a maximal F_β_ score of 0.9. NanoPrePro identified the highest F_β_ score from all the maximal F_β_ scores and selected the parameter combinations that achieved this highest value as the final and optimal cutoffs. In our demonstration, the highest maximal F_β_ score was 0.9. Therefore, the parameter combinations that resulted in this maximal F_β_ score of 0.9 were designated as the cutoff by NanoPrePro.

We equipped NanoPrePro with a novel algorithm that overcomes the challenge of distinguishing true alignments from random ones by simulating both types of alignments and determining the optimal cutoff to differentiate them (Fig. 2C). In contrast, the algorithm in Pychopper maximizes the output read number rather than minimizing the random alignments (Fig. 2B; Fig. S2). The algorithm of NanoPrePro first simulates true and random AP alignments by performing alignment twice on each read (Fig. 3A). This approach is based on the ONT library construction kit design, where a 5′ or 3′ adapter/primer should be found exactly once on the reads (Fig. S1). Thus, the first alignment with higher sequence similarity is regarded as the true alignment (Fig. 3A, top), and the second alignment, performed while masking the regions of the first alignment, serves as the random alignment (Fig. 3A, bottom).

True alignments are expected to be located within the first or last several bases on the reads and exhibit high similarity to the theoretical AP sequences (examples for locating on first-several bases, blue samples in Fig. 3A and B). Conversely, random alignments can occur at any other location and typically show relatively low similarity to the theoretical sequences (orange samples in Fig. 3A and B). Based on the distribution of true and random alignments, NanoPrePro determines the cutoffs on sequence similarity and location to distinguish them (the red dotted lines in Fig. 3B).

NanoPrePro evaluates the effectiveness of the cutoffs using the F_β_ score, which is the harmonic mean of two metrics: precision and recall. Precision measures the ability to exclude random alignments, and recall assesses the ability to retain true alignments. Among all possible combinations of sequence similarity and location, NanoPrePro selects the ones that maximize the F_β_ score, leading to the highest precision and recall, and sets these as the cutoffs (the red dotted lines in Fig. 3B).

Besides sequence similarity and location, another critical factor for alignment is the AP length (later as the length of the AP substring) used for alignment. Because ONT sequencing suffers lower quality toward read terminals (Fig. S4B) [14, 35], longer AP substrings lead to lower sequence similarity for true alignment, on the other hand, shorter AP substrings cause higher sequence similarity for random alignment (Fig. 3C). As mentioned above, NanoPrePro obtains the best F_β_ score based on sequence similarity and location for a fixed AP length. Different AP lengths generate different “best F_β_ scores”, and the corresponding sequence similarity, location, and length of AP substring from the highest of these “best F_β_ scores” are used as the cutoff (Fig. 3C). Thus, true AP alignments are recognized by NanoPrePro as the alignment results of an “optimal length of AP substring” that passes both “location” and “sequence similarity” cutoffs (Fig. 3C).

Based on the identified APs, NanoPrePro then performs the five preprocessing tasks (Fig. 1E). Full-length reads are characterized by the presence of 5′ and 3′ APs, while reads missing either APs (or both) are characterized as truncated reads. Chimeric reads are recognized by the alignments of APs to the center of the reads, which are the alignment results passing the sequence similarity cutoff but failing the location cutoff (indicated by black arrows in Fig. 3B). AP sequences and the adjacent polyA/T sequences are trimmed based on the pinpointed location of the APs. Reorientation by strand is performed based on the strand of the identified AP (Fig. S1F). Finally, quality filtering will be carried out as the last step of preprocessing by NanoPrePro, which provides a better assessment of the read quality without the perturbation of low-quality terminal bases.

### NanoPrePro’s performance in identifying full-length reads

The precise identification of full-length reads is the highest priority in NanoPrePro’s algorithm design, given the negative impact on downstream analyses from falsely classifying chimeric or truncated reads as full-length. For benchmarking, NanoPrePro was compared with Pychopper in identifying full-length reads from 63 simulated datasets consisting of varying proportions of full-length, chimeric, and truncated reads with different read quality. Among the simulated datasets, the proportion of full-length reads ranged from 50% to 100%, fusion reads ranged from 0% to 10%, and truncated reads ranged from 0% to 50% (Table S3). Reads of varying quality were simulated with error rates of ~14%, 5%, and 0.1% (Table S3). We then compared the performance of NanoPrePro and Pychopper using these 63 simulated datasets.

**Figure 4:**
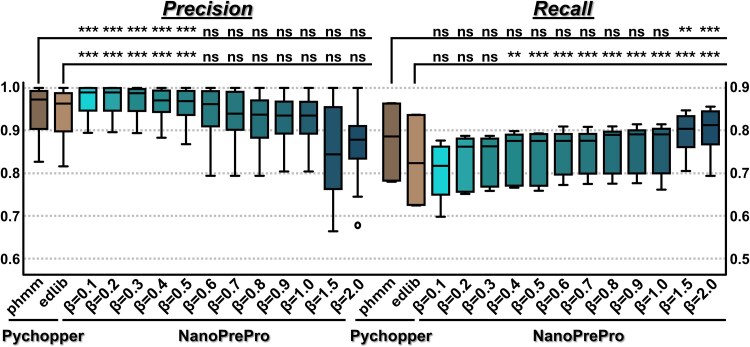
The performance of full-length read identification by Pychopper and NanoPrePro. The boxplots showed the precision (left panel) and recall (right panel) for Pychopper and NanoPrePro in identifying full-length reads from 63 simulated datasets. The y-axis in the left panel displayed precision values, while the y-axis in the right panel displayed recall values. The performance of Pychopper, using phmm and edlib backends, was shown in dark and light brown, respectively. NanoPrePro results across β values ranging from 0.1 to 2.0 are shown in a turquoise color scheme. “^***^” indicated *P* < .001, “^**^” indicated *P* < .01, and “ns” indicated not significant in the Wilcoxon signed-rank test (one-tailed).

Different users may have varying needs regarding correctness (precision) or yield (recall). Precision calculates the ratio of actual full-length reads to all reads identified as full-length, and recall calculates the ratio of retrieved full-length reads to all full-length reads in a dataset. The β factor in F_β_ scores adjusts the weighting between precision and recall, with a lower β placing more emphasis on precision. We examined the performance of NanoPrePro under different weighted precision and recall using β factors ranging from 0.1 to 2.0 (Fig. 4; Fig. S3 showing PR and ROC curves). NanoPrePro exhibits higher precision when β values are lower and higher recall when β values are higher, a behavior consistent with the expected trade-off between precision and recall. This indicates that the algorithm responds to parameter tuning in a predictable and interpretable manner, consistent with well-established precision–recall dynamics. When precision is regarded as more important than recall, or even as a prerequisite to recall, we observed that NanoPrePro maintains consistently high precision when β values are set between 0.1 and 0.3 (Fig. 4). We therefore used this optimal range for NanoPrePro and compared its performance to that of Pychopper, run with the phmm and edlib backends, in identifying full-length reads (Fig. 4).

NanoPrePro (β = 0.1/0.2/0.3) achieved median precision values around 0.99, which were significantly higher than Pychopper running on either backend (medians of 0.97 and 0.96 for phmm and edlib, respectively) as tested using the Wilcoxon signed-rank test (Fig. 4). Such an improvement in precision was achieved at the cost of lower recall rates of full-length reads, as we observed that NanoPrePro showed significantly lower or similar recall rates compared to Pychopper running on phmm or edlib backend, respectively (Fig. 4). This drawback could be easily counterbalanced by increasing sequencing throughput, as the cost of sequencing is dramatically decreasing. Noticeably, for Pychopper, the phmm backend outperformed edlib in both precision and recall. Thus, we excluded Pychopper running on the edlib backend in further analyses. Given the absolute and unparalleled importance of eliminating false positives from downstream analyses, NanoPrePro outperformed Pychopper by delivering significantly higher precision in full-length read identification.

### NanoPrePro’s performance on read preprocessing tasks

To compare NanoPrePro’s performance on read preprocessing with Pychopper using real data, we performed a total of seven ONT sequencings for three economically important plant species (*Populus trichocarpa*, *Eucalyptus grandis,* and *Liriodendron chinense*) (Table S4), and downloaded two datasets from published human cancer data. The sequencing libraries were constructed using ONT sequencing kits SQK-PCS109 and SQK-PCS111, which are both PCR-based sequencing kits that are optimized for transcriptome profiling and higher throughput. To facilitate the following analyses and normalize different throughputs, 100 000 reads were randomly sampled from each ONT transcriptome dataset. The average error rates of the sampled reads ranged from 7% to 10% (Table S5). From the average Q-score of all raw reads (Fig. S4A), the average Q score of each base in each read (Fig. S4B), and read length (Fig. S4C), our results from seven libraries showed high consistency with the published two human cancer libraries, demonstrating the robustness of our pipeline with the published data.

**Figure 5:**
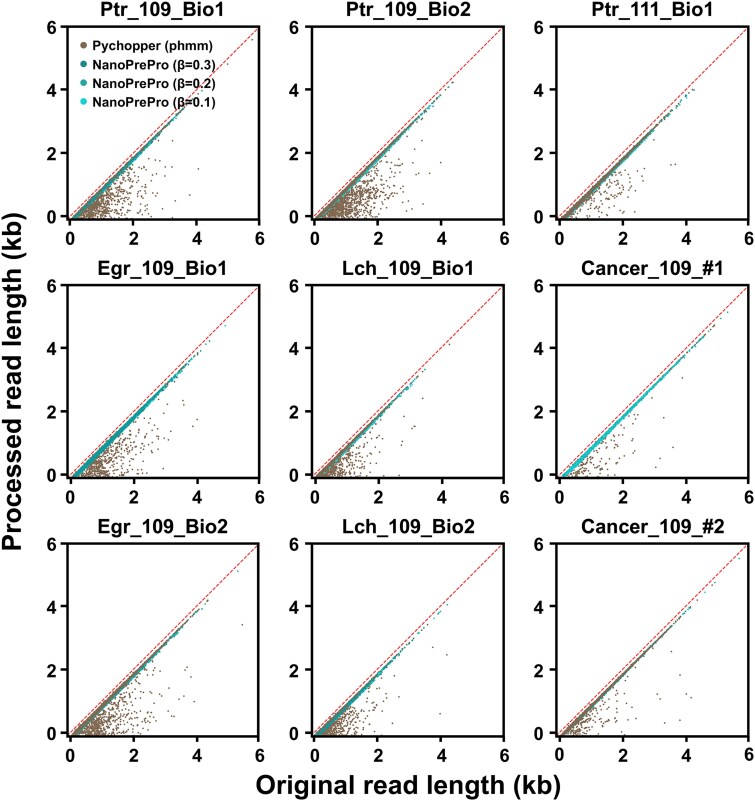
The lengths of the full-length reads identified and preprocessed by Pychopper and NanoPrePro across nine ONT transcriptomic datasets. Each dot on the scatter plot represented a full-length read identified by Pychopper (using phmm backend, shown in brown) or NanoPrePro (with factors β = 0.1/0.2/0.3 shown respectively in light turquoise, medium turquoise, and dark turquoise). The x-axis showed the original length (kb, kilobase) of identified full-length reads before preprocessing, while the y-axis displayed the length after being preprocessed by Pychopper or NanoPrePro. The red-dotted lines represented the function of x = y. Ptr, *P. trichocarpa*; Egr, *E. grandis*; Lch, *L. chinense*. 109, SQK-PCS109; 111, SQK-PCS111. Bio, biological replicate.

In total, nine datasets were processed by NanoPrePro and Pychopper to identify and preprocess full-length reads. To demonstrate the effect of the β values on cutoff selection, we compared the average values of the three alignment cutoffs under varying β values using eight SQK-PCS109 datasets. Among the three cutoffs, the location cutoff remained relatively stable, whereas the balance between precision and recall was mainly adjusted through the similarity cutoff and AP length (Table S6). Comparing the full-length reads identified and preprocessed by NanoPrePro and Pychopper, we found that many full-length reads generated by Pychopper were noticeably shorter compared to their unprocessed length (Fig. 5 and Table S7). Such abnormality may be caused by Pychopper’s algorithm for extracting the maximal number of full-length reads, which could split reads in the middle while scanning for potential APs across the entire reads. Although the percentage is relatively low (0.5%–3.0%), such abnormalities represent thousands of reads as false results and would be included in downstream analyses. This presents a problem with Pychopper that could be avoided by using NanoPrePro.

Processed reads were mapped to the reference genome to assess NanoPrePro and Pychopper’s performance on preprocessing. Sampled ONT reads, referred to as raw reads, were also mapped to the reference genome as controls. By comparing the number of soft-clipped bases, the leading and trailing bases on a read that do not match the reference genome, we observed that both NanoPrePro and Pychopper significantly reduced the artifacts on the processed reads compared to raw reads (Fig. S5). In addition, NanoPrePro showed improvement over Pychopper by significantly minimizing the number of soft-clipped bases (Fig. S5).

For reference-based transcriptome analysis, artifacts on reads can be removed through soft-clipping, but this approach is unavailable to species without a reference genome. Reference-free transcriptome analyses are fitted for long-read technologies, because long-reads could potentially cover the entire transcript in one read. For reference-free analyses, we performed reference-free transcriptomic reconstruction using NanoPrePro and Pychopper preprocessed full-length reads, and mapped the reconstructed transcripts to the reference genome to assess the effect of those unmappable artifacts (residual AP sequences or polyA/T) on the analyses. We observed that the artifacts on reads were incorporated and magnified in reconstructed transcripts. NanoPrePro showed improvement over Pychopper by significantly reducing these artifacts in reconstructed transcripts (Fig. S6). Moreover, reconstructed transcripts were compared with the annotation to evaluate the completeness of the transcripts. We observed a 0%–3% elevation in median transcript coverage while using full-length reads generated by NanoPrePro, and the differences were significant in *Populus* and *Eucalyptus* (Fig. S7). The improved transcript coverage may result from NanoPrePro’s higher precision in full-length read identification and its design to avoid splitting reads in the middle, which both prevent residual AP sequences and truncated reads from participating in transcript reconstruction.

### Run-time and memory consumption

Finally, we compared NanoPrePro and Pychopper on resource consumption, which is another critical aspect of benchmarking, especially with the growing throughput of modern ONT sequencing. Two traits in Pychopper’s algorithms lead to longer run times and higher memory consumption compared to NanoPrePro. First, Pychopper applies a “profile hidden Markov model” for AP alignment, which exhibits a higher time complexity than the “Hirschberg’s algorithm” used by NanoPrePro [54, 55]. Second, intending to rescue chimeric reads, Pychopper marks every potential APs across the entire reads and applies a dynamic programming algorithm to find the best configuration of AP pairs that maximizes the output read number. In contrast, NanoPrePro exerts a straightforward read-type classification algorithm (full-length, truncated, and chimeric).

**Figure 6:**
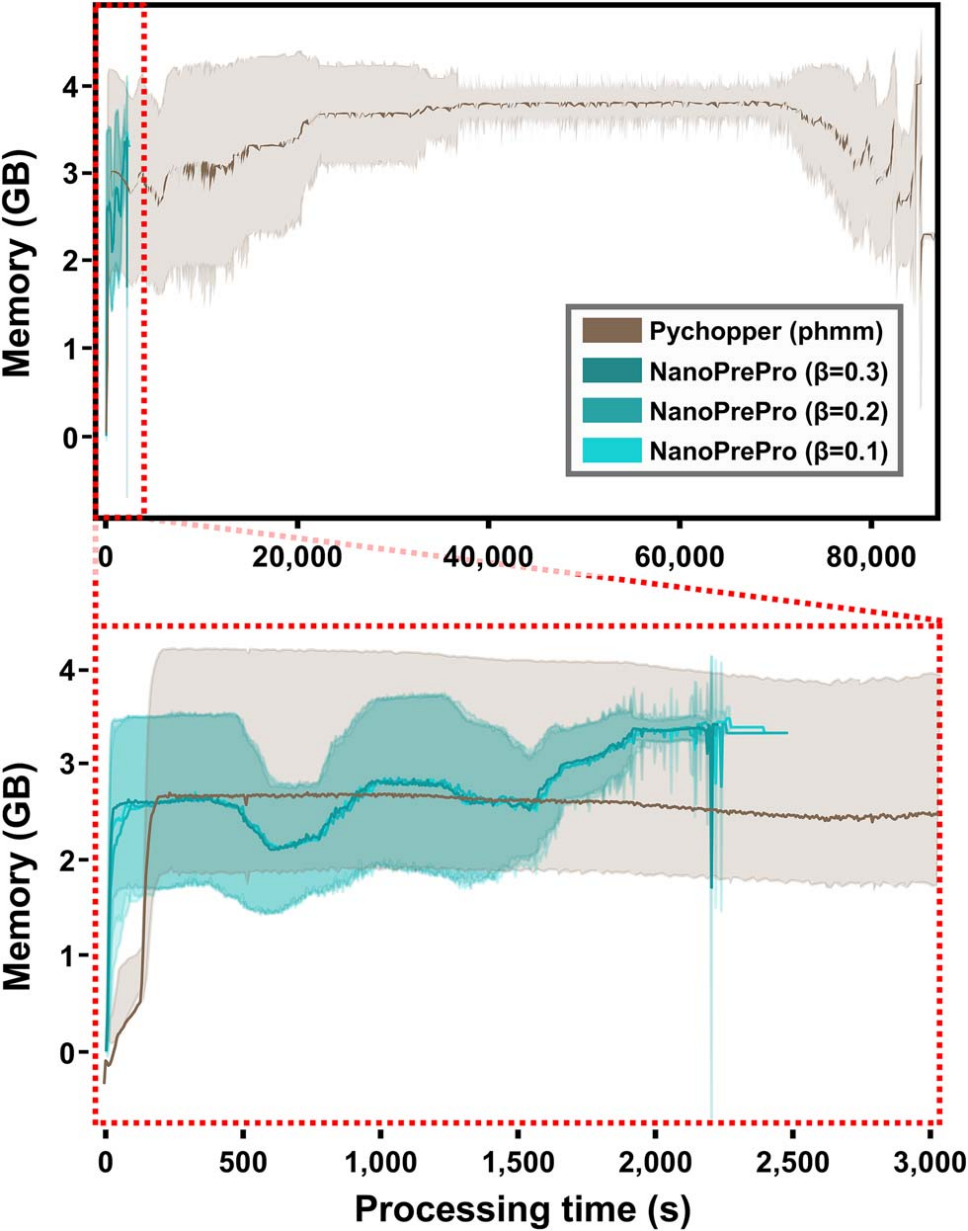
Memory consumption of NanoPrePro and Pychopper over time. The diagram showed the average computer memory consumption over time by Pychopper and NanoPrePro while processing a total of 72 datasets (63 simulated and 9 real ONT transcriptomic datasets), each with 100 000 reads. Pychopper, using the phmm backend, was shown in brown. NanoPrePro with factors β = 0.1/0.2/0.3 were shown respectively in light turquoise, medium turquoise, and dark turquoise. The lighter-colored background indicated the standard deviation of memory consumption. The bottom panel was a zoomed-in view of the area circled by red dotted lines on the upper panel, which provided a clearer illustration of the detailed memory consumption of Pychopper and NanoPrePro. GB, Gigabyte; s, second.

We compared NanoPrePro’s resource consumption with Pychopper by measuring the run-time and memory consumption of processing the aforementioned simulated datasets and real ONT transcriptome datasets. NanoPrePro demonstrated substantial improvement over Pychopper in resource consumption by requiring only 1/38 of the run-time and using less memory (Fig. 6).

### NanoPrePro performance on the latest and early-access kits

There are three types of the latest or early-access V14 sequencing kits: ligation sequencing (LSK114), cDNA-PCR sequencing (PCS114), and direct RNA sequencing (RNA004).

For ONT sequencing, basecallers such as Guppy and Dorado can also function as preprocessors, with preprocessing steps that may be either optional or enforced depending on the workflow. For cDNA sequencing (LSK114 and PCS114), users can turn off AP removal in the basecaller and instead apply alternative preprocessors such as NanoPrePro or Pychopper, which provide more comprehensive preprocessing than the basecaller’s built-in functions (Table S2). Regarding direct RNA sequencing (RNA004 or earlier versions), ONT’s current basecalling software (Dorado) enforces AP removal during basecalling, and this function cannot be disabled. Moreover, direct RNA sequencing involves adapter ligation at only one end of the RNA molecule, which makes its adaptor removal logic fundamentally different from that of cDNA sequencing (AP on both ends). Therefore, neither Pychopper nor NanoPrePro can support direct RNA sequencing data.

For the ligation sequencing kit (LSK114), we evaluated two published ONT datasets (Fig. S8A–C): single-cell transcriptomic sequencing of the 293T cell line and spatial transcriptomic sequencing of the mouse brain. Compared with Pychopper, NanoPrePro more effectively avoided over-segmentation (Fig. S8D) and performed significantly better in removing AP sequences on processed reads and reconstructed transcripts (Fig. S8E and F). Furthermore, to quantitatively assess the contribution of higher preprocessing precision to transcriptome reconstruction, we evaluated isoform-level recall and BUSCO completeness. NanoPrePro retrieved more transcript isoforms (Fig. S8G) and recovered more BUSCO markers (Fig. S8H) than Pychopper in both datasets. The most striking difference was observed in BUSCO completeness from the spatial transcriptomic dataset (LSK114 Spatial, Fig. S8H), where Pychopper recovered fewer than half the markers retrieved by NanoPrePro.

For the cDNA-PCR sequencing kit (PCS114), we analyzed a published bulk transcriptomic dataset of the mouse retina (Fig. S8A–C). This kit is currently available as an “early access” product undergoing final validation and stability testing by ONT (ONT website, accessed 25 August 2025) and is not supported by Pychopper (v2.7.10). We tested NanoPrePro on two data subsets and found consistent improvements in avoiding over-segmentation (Fig. S8D) and achieving better AP removal (Fig. S8E and F), underscoring the effectiveness of the PCS114 kit in combination with NanoPrePro for recovering complete transcripts.

Among the LSK114 and PCS114 samples, notable differences were observed between the bulk and spatial transcriptomic datasets. The spatial transcriptomic dataset (LSK114 Spatial; Fig. S8G and H) yielded the largest number of transcript isoforms and BUSCO markers, reflecting the higher spatial resolution of this technology, which captures diverse cellular states across tissue regions. However, this dataset also contained a higher proportion of incomplete isoforms and fragmented BUSCO markers, primarily due to RNA degradation during sample preparation. These issues are intrinsic to the spatial transcriptomic workflow and reflect data-quality limitations rather than any deficiency in NanoPrePro’s processing performance. In contrast, the bulk dataset (PCS114) showed a higher proportion of full-length isoforms and intact BUSCO markers (PCS114 Bulk_#1 and #2; Fig. S8G and H), consistent with its higher RNA integrity. Together, these findings demonstrate the forward compatibility of NanoPrePro with data from the latest ligation and cDNA-PCR sequencing kits (LSK114 and PCS114) and highlight its flexibility in preprocessing both standard bulk libraries and non-standard libraries generated by combining single-cell or spatial transcriptomic protocols with the LSK114 workflow.

### Consistent cutoff stringency via self-optimizing algorithm

During basecalling, ONT provides three model options: “sup,” “hac,” and “fast,” which represent the tradeoff between accuracy and processing speed. The “sup” model achieves the highest accuracy, whereas the “fast” model runs the quickest. To examine how basecalling models affect NanoPrePro’s cutoff selection, we applied different models to datasets generated with multiple kit versions. Across a β range of 0.1–2.0 (Fig. S9), while comparing the effects of kit version and basecalling model, the kit version has a greater influence on the optimal cutoff values (similarity, location, and AP length) than the basecalling model. When examining the effect of the basecalling model alone, higher accuracy models generally allow the use of more stringent cutoffs. With the sup model (more accurate model), NanoPrePro tends to apply more stringent cutoff on similarity and location (higher similarity cutoffs and narrower location cutoffs) for AP alignment. Namely, AP sequences must achieve higher sequence similarity and position more precisely on the read (less tolerance to insertion or deletion). In contrast to similarity and location cutoffs, AP length cutoff showed an opposite trend (less stringent), which is likely a compensatory effect that a shorter AP length (less stringent) helps balance precision and recall under the stricter similarity and location cutoff. These results are consistent from analyzing different data sets (Table S6 and Fig.  S9).

These results highlight the advantage of NanoPrePro’s self-optimizing algorithm. Compared to existing strategies that rely on fixed cutoffs and non-adaptive rules, often failing to maintain consistent stringency across datasets, our approach achieves comparable stringency automatically and with greater robustness. Namely, a similarity cutoff of 0.7 may be overly strict for older kits but too permissive for newer kits with higher sequencing accuracy. By adapting cutoffs through F_β_-based optimization, NanoPrePro ensures a consistent precision–recall tradeoff across diverse datasets, providing a robust and generalizable framework for transcriptome preprocessing.

## Discussion

We present NanoPrePro, a new preprocessor for ONT sequencing data that was equipped with all essential preprocessing functions and applied a novel algorithm to optimize adapter/primer identification parameters for each sequencing file. Compared to the state-of-the-art preprocessor Pychopper, NanoPrePro demonstrated considerable improvement by providing higher precision in full-length read identification and significantly reducing biases for downstream analyses while requiring less run-time and computer memory.

### Dependence of NanoPrePro on Oxford Nanopore Technologies sequencing quality

NanoPrePro’s algorithm was based on the ideal scenario that an AP sequence will align perfectly once to a read, which is not the case for truncated and chimeric reads. Truncated reads make AP sequences align less than once, while chimeric reads make multiple alignments. Using simulated data, we have demonstrated that NanoPrePro could work with as few as only 50% of full-length reads in a dataset (Table S3), which is much lower than the percentage expected in current ONT sequencing. Namely, full-length reads, which constitute the majority, enable NanoPrePro to identify the optimal parameters. Furthermore, with the anticipated improvements from ONT, which will align more closely with the assumptions underlying NanoPrePro’s algorithm, we expect NanoPrePro’s performance to improve accordingly.

### NanoPrePro requires much less processing time

To meet the increased need for sequencing throughput, ONT launched GridION and PromethION sequencing platforms to sequence up to 5 or 48 flow cells in parallel (https://nanoporetech.com/community). The throughput from each flow cell has also been upgraded. With the current boost of throughput, the downstream processing time dramatically increased. In this study, the average throughput increased around two times with the upgrade from SQK-PCS109 to SQK-PCS111 (Table S4). For the analysis of 100 000 reads, the preprocessing times are ~40 and 1500 min by NanoPrePro and Pychopper, respectively, where NanoPrePro demonstrates a processing speed 38 times faster than Pychopper (Fig. 6). The total throughput of SPK-PCS111 sequencing kits is 31.7 million reads (Table S4), which will take 330 days (1500 × 317 min) for Pychopper to process the entire dataset on one thread. In contrast, it will take only 8.8 days (40 × 317 min) for NanoPrePro to finish the task.

### NanoPrePro in facilitating reference-free transcriptomic studies

Many studies skipped the preprocessing steps and directly mapped the raw reads to reference genomes or transcriptomes for the subsequent analyses [3, 56]. However, preprocessing involves many critical steps, and mapping to the reference genome can only execute AP region removal by soft clipping and quality filtering by the mapping capability [3]. Ignoring the other critical steps would cause numerous problems mentioned above (see Introduction). For example, without the strand-specific information, the quantification of transcript abundance and characterization of exon-intron boundaries would suffer from data misinterpretation.

Currently available reference genomes can be categorized into four groups based on their assembly levels from low to high as Contig, Scaffold, Chromosome, and Complete Genome. Mapping to reference genomes with higher assembly levels can further polish or replace the two preprocessing steps, AP region removal and quality filtering. However, without performing all preprocessing steps, mapping to the reference genome would still lead to incorrect loci mapping, especially using low assembly-level reference genomes. In addition, high assembly level reference genomes, such as Complete Genome level, are only 1.3% and 12.9% of all available eukaryotic and prokaryotic reference genomes, respectively (Table S8). So far, around 1.22 million eukaryotic species have been identified, and around 8.7 million species are expected to exist worldwide [57], which means that the species with available genome information are extremely scarce. The low percentage of available genome information among species and the low percentage of high assembly-level reference genomes severely hinder the applicability of the strategy of using genome mapping to replace partial preprocessing steps.

### Balancing recall and precision through parameter optimization

Different users may prioritize either recall or precision, and the choice of β factors becomes an important parameter in optimizing NanoPrePro’s performance. Higher β values emphasize recall, while lower β values emphasize precision. A higher β increases the recovery of full-length reads but may also include more truncated reads, which could ultimately reduce effective recall. Conversely, a lower β improves precision by enforcing stricter criteria but may sacrifice yield. Although Pychopper may report higher recall values under certain parameter settings, such outcomes must be interpreted with caution. Higher recall in the absence of sufficient precision does not reflect genuine recovery of full-length reads but rather the inclusion of false positives, which can mislead downstream analyses. This apparent gain in recall is therefore potentially artificial and detrimental. In fact, increasing recall in NanoPrePro would be very easy and straightforward. For example, by including truncated reads or lowering precision thresholds, but such practices would compromise data reliability. By contrast, we made NanoPrePro prioritize precision, ensuring that classified reads are reliable before any attempt is made to optimize recall. We emphasize that meaningful improvements in recall are only valuable when built upon a foundation of high precision, and users should be cautious not to overinterpret inflated recall metrics lacking this reliability.

The β factors in NanoPrePro primarily function to coordinate three AP-related cutoff parameters: length, location, and similarity. We found that location cutoff exhibits highly stable values across different β factors within the same kits (Table S6), indicating that β factor adjustment primarily affects the AP length and similarity cutoffs. Stringent cutoffs (longer AP lengths and higher similarity cutoffs) favor precision, whereas relaxed cutoffs (shorter AP lengths and lower similarity cutoffs) favor recall. These principles serve as general guidelines for users who wish to manually adjust cutoff parameters. In general, when aiming to increase recall, both similarity and AP length cutoffs tend to be relaxed, which can lead to an imbalance between recall and precision. NanoPrePro addresses this by simultaneously considering both cutoffs, allowing fine-tuned adjustments so that prioritizing recall does not completely compromise precision, and vice versa. Specifically, NanoPrePro applies relaxed similarity thresholds in combination with stringent AP length cutoffs when the F_β_ scores are weighted toward recall, and stringent similarity thresholds with relaxed AP length cutoffs when the scoring favors precision. This coordinated adjustment highlights the strength of NanoPrePro’s self-optimizing algorithm in jointly optimizing multiple parameters to achieve balanced and robust performance.

To facilitate user manual adjustments, we provide an interactive HTML report generated by NanoPrePro for users to identify their ideal outcomes using F_β_-score optimization (Fig. S10). In addition, detailed guidelines are also provided in the online documentation (ReadTheDocs), including information on installation, implementation, and output.

### NanoPrePro forward compatibility

ONT sequencing can be considered the most widely used platform to obtain long-read transcriptomic data. Increasing base quality and sequencing throughput are the two major developing directions of ONT sequencing by the improvement of library construction kit chemistry, nanopores on the flow cells, sequencing devices, and new versions of basecallers. For example, an upcoming upgrade of library construction kits and nanopores will enhance base quality and bring read quality scores up to Q20 (https://nanoporetech.com/community). Plongle is a new sequencing device in development for a throughput scale-up to process 96 flow cells at one time. Based on our design, NanoPrePro can easily achieve forward compatibility for the foreseeable upgraded version of ONT transcriptomic sequencing. For base quality, more accurate base information will allow NanoPrePro to identify AP regions more accurately. For sequencing throughput, if Plongle is used to process results from 96 flow cells, NanoPrePro would require ~845 days (96 × 8.8 days), based on the processing time of Ptr_111_Bio1 for one flow cell using a single thread. In contrast, Pychopper would need nearly 87 years (96 × 330 days). This long processing time can be partially reduced by increasing the number of threads. With the latest Intel server-grade CPUs capable of supporting up to 144 threads, NanoPrePro would need only ~5.9 days, while Pychopper would still require around 220 days. This difference demonstrates a substantial gap between the two software. Given that not all research facilities have access to such high-performance computing equipment, the extended computing times with Pychopper could still pose challenges for some researchers, particularly in handling extensive data volumes.

One of the spirits of ONT is its portable characterization. ONT is also developing SmidgION, a portable sequencing device operated by a smartphone to meet the needs of field-based research such as real-time species identification in food, timber, or wildlife. During the whole procedure of transcriptomic sequencing, a read would be regarded as a “usable read” after completing the preprocessing steps. Thus, a portable preprocessor installed on a smartphone can provide on-site evaluation for the users to determine whether the usable reads are enough for their subsequent analyses. Compared to workstations or even laptops, smartphones hold much less memory. NanoPrePro requires less memory cost (Fig. 6), which is more suitable to be adapted for smartphone applications. In summary, NanoPrePro possesses the ability of forward compatibility and can also co-evolve with the upgrades of ONT.

**Key Points**

- NanoPrePro integrates six essential functions for preprocessing Oxford Nanopore sequencing data.
- NanoPrePro requires less processing time and memory compared to existing tools.
- NanoPrePro maintains forward compatibility with both current and early-release sequencing kits.
- NanoPrePro supports datasets combining single-cell and spatial protocols with ONT sequencing technologies.
- NanoPrePro self-optimizing algorithm ensures consistent stringency across different kits and basecall models.

## Supplementary Material

Supplementary_Materials_bbag063

Supplementary_Methods_bbag063

## Data Availability

NanoPrePro is available on GitHub (https://github.com/Woodformation1136/NanoPrePro). Raw signal files for plant ONT transcriptomic data are available at the Zenodo archive (10.5281/zenodo.17304252). The Zenodo archive also stores a mirror version of the NanoPrePro repository. Human ONT transcriptomic data used in this study are accessible at NCBI SRA (SRR14326970 and SRR14326973) [58]. Mouse retina transcriptomic data are accessible at NCBI GEO (GSE255520) [59].

## Notes

Biographical note.

The authors are a collaborative research team from National Taiwan University and National Yang Ming Chiao Tung University, with research interests ranging from xylem development to anticancer drug research.
