# Supplementary material for: NanoPrePro: a fully equipped, fast, and memory-efficient preprocessor for nanopore transcriptomic sequencing
Source: Brief Bioinform. 2026 Feb 13;27(1):bbag063. doi: 10.1093/bib/bbag063 (PMC12903951; doi:10.1093/bib/bbag063)
Supplement: Supplementary_Materials_bbag063 [file supplementary_materials_bbag063.pdf]

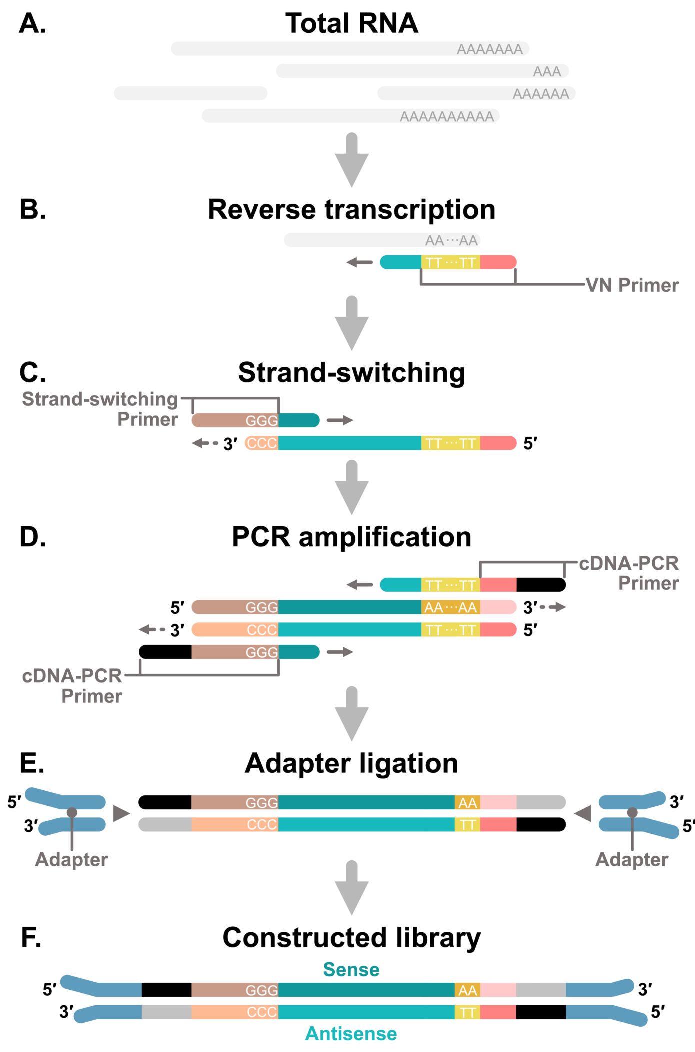

Figure S1. The workflow for PCR-based ONT transcriptomic sequencing library construction. (A) Library preparation for ONT transcriptomic sequencing started with total RNA. **(B)** The polyadenylated RNA was purified by VN primer, and complementary DNA (cDNA) was then generated by reverse transcription. **(C)** The second strand of the cDNA was synthesized through strand switching using a strand-switching primer. **(D)** PCR was performed to amplify the cDNA library with cDNA-PCR primers. **(E)** Adapters were ligated to both ends of the PCR products. **(F)** In the final constructed library, the strands of fragments representing the original transcripts are defined as sense strands, while their complementary strands are defined as antisense strands.

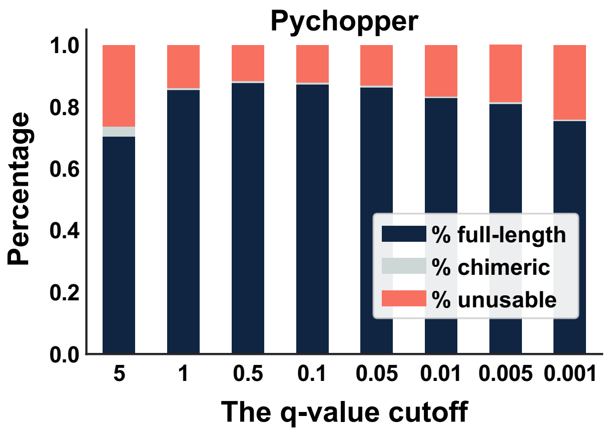

Figure S2. Proportions of full-length, chimeric, and unusable reads obtained by Pychopper under different q-value cutoffs. Pychopper (version 2.7.10) was applied to the dataset Egr_109_bio1, with q-value cutoffs ranging from 5 (relaxed) to 0.001 (stringent). These cutoffs were specified using the "-q" parameter, which indicates the statistical significance of the alignment. A cutoff of 5 corresponds to an expectation of an average of five false-positive adapter/primer alignments per read. Full-length, chimeric, and unusable reads are shown in dark blue, light grey, and coral, respectively.

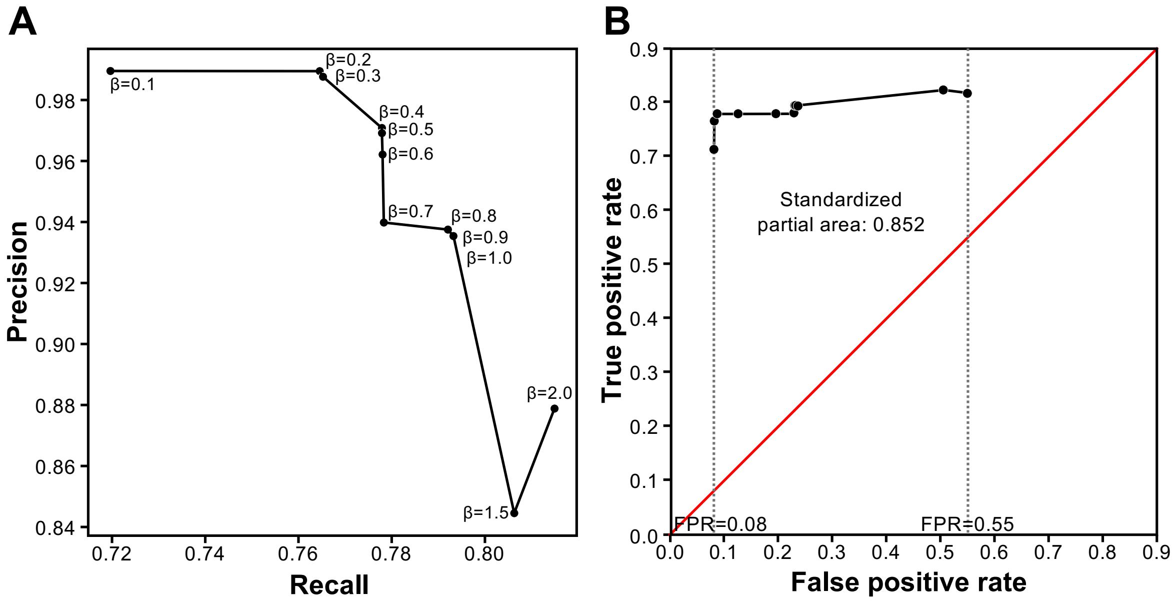

Figure S3. The PR curve, ROC curve, and the corresponding AUC score of NanoPrePro. (A) The Precision–Recall (PR) curve shows the median recall and precision for identifying full-length reads across 63 simulated datasets under different β settings (β = 0.1–2.0). Recall is the proportion of full-length reads that are correctly retrieved, and precision is the proportion of classified full-length reads that are truly full-length. **(B)** The receiver operating characteristic (ROC) curve shows the median false positive rate and true positive rate for identifying full-length reads across 60 simulated datasets under varying β settings (β = 0.1–2.0). Three datasets containing only full-length reads were excluded because false positive rate cannot be computed for such datasets. False positive rate (FPR) is the proportion of non–full-length reads incorrectly classified as full-length. True positive rate is equal to recall and is defined as described above. The standardized partial area under the ROC curve (McClish, 1989) was calculated within the range of FPR = 0.08–0.55. The red line represents the ROC curve of a random classifier, which yields a diagonal line with equal false positive and true positive rates across all thresholds.

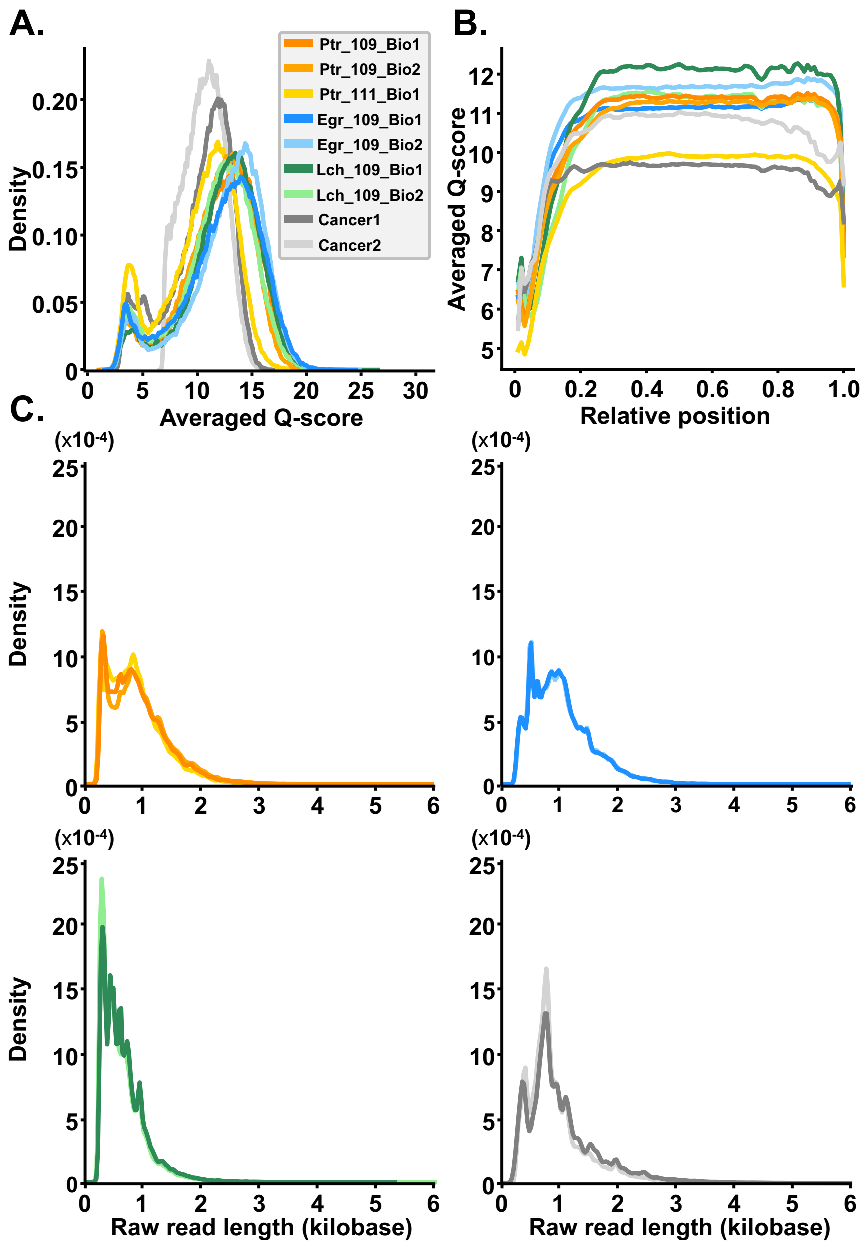

Figure S4. The distribution of read length and quality in ONT transcriptomic datasets. (A) The distribution of the average Q-score is visualized using kernel density estimation, with the x-axis representing the average Q-score and the y-axis representing the estimated density. **(B)** The average Q-score was calculated at each relative position on the reads, where 0 corresponded to the first base and 1 corresponded to the last base. **(C)** The distribution of read lengths was visualized using kernel density estimation, with the x-axis representing read length and the y-axis representing estimated density. The species related to Ptr was shown in the top-left, Egr in the top-right, Lch in the bottom-left, and human cancer in the bottom-right. Brown, Ptr_109_Bio1; Orange, Ptr_109_Bio2; Yellow, Ptr_111_Bio1; Dark blue, Egr_109_Bio1; Light blue, Egr_109_Bio2; Dark green, Lch_109_Bio1; Light green, Lch_109_Bio2; Dark grey, Cancer_109_#1; Light grey, Cancer_109_#2. Ptr, *P. trichocarpa*; Egr, *E. grandis*; Lch, *L. chinense*. 109, SQK-PCS109; 111, SQK-PCS111. Bio, biological replicate.

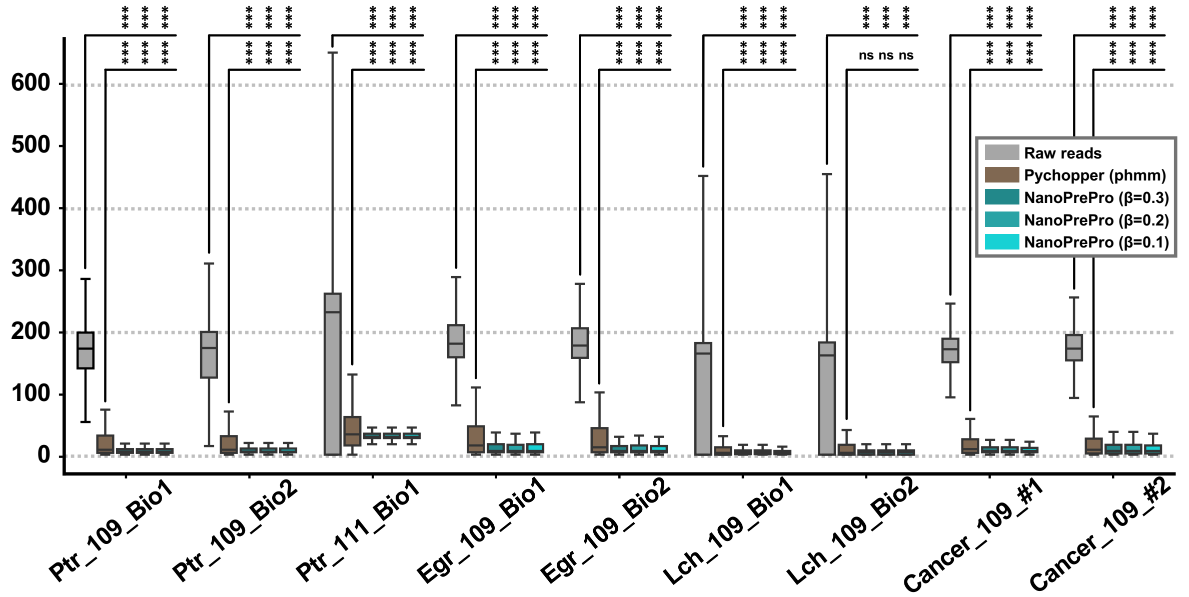

Figure S5. The number of soft-clipped bases of Pychopper and NanoPrePro pre-processed reads. Full-length reads were identified and pre-processed by Pychopper and NanoPrePro from 9 real ONT transcriptomic datasets, with the raw reads serving as unprocessed controls. The number of soft-clipped bases was summarized after mapping the full-length reads to the reference genome, and the data were visualized using boxplots. The soft-clipped bases from the raw reads were shown in grey. The performance of Pychopper (brown), using the phmm backend, was presented alongside the results of NanoPrePro (turquoise), with factors beta = 0.1, 0.2, and 0.3. "***" indicated $p < 0.001$ in Dunn's test, with the false discovery rate controlled using the Benjamini-Hochberg method. Ptr, *P. trichocarpa*; Egr, *E. grandis*; Lch, *L. chinense*. 109, SQK-PCS109; 111, SQK-PCS111. Bio, biological replicate.

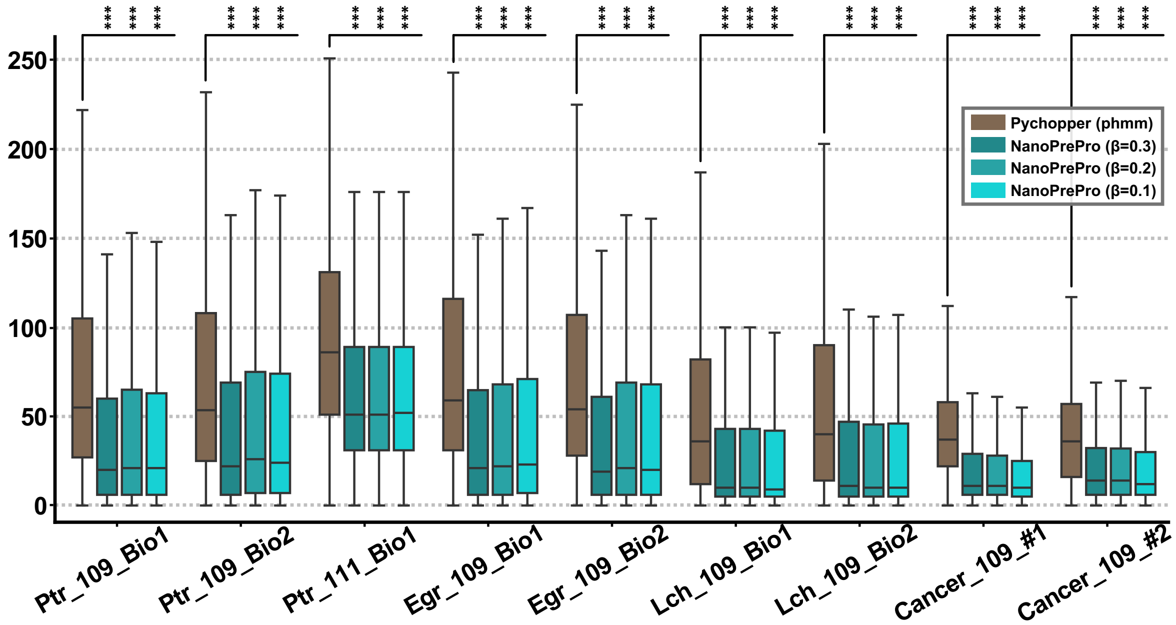

Figure S6. Differences in the number of soft-clipped bases in reconstructed transcripts between Pychopper and NanoPrePro. The number of soft-clipped bases was summarized after mapping the reconstructed transcripts, generated using full-length reads from Pychopper and NanoPrePro, to the reference genome. Pychopper, using the phmm backend, was shown in brown. NanoPrePro, with factors beta = 0.1, 0.2, and 0.3, was displayed in light turquoise, medium turquoise, and dark turquoise, respectively. "***" indicated p < 0.001 in Dunn's test, with the false discovery rate controlled using the Benjamini-Hochberg method. Ptr, *P. trichocarpa*; Egr, *E. grandis*; Lch, *L. chinense*. 109, SQK-PCS109; 111, SQK-PCS111. Bio, biological replicate.

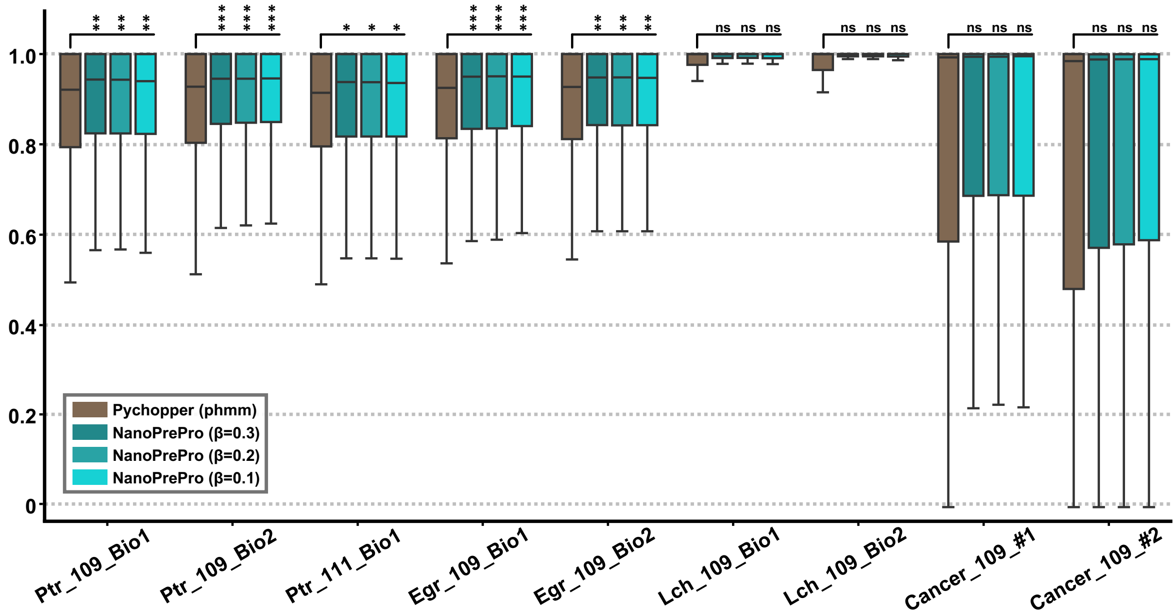

Figure S7. Comparing transcript coverage of reconstructed transcripts from Pychopper and NanoPrePro. Transcript coverage was assessed by mapping the reconstructed transcripts, derived from full-length reads using Pychopper and NanoPrePro, to the reference genome. The coverage ratio for each reconstructed transcript was calculated based on the best-matched reference transcript isoform. Pychopper, using the phmm backend, was shown in brown. NanoPrePro, with factors beta = 0.1, 0.2, and 0.3, was displayed in light turquoise, medium turquoise, and dark turquoise, respectively. "***" indicated $p < 0.001$, "**" indicated $p < 0.01$, "*" indicated $p < 0.05$, and "ns" indicated not significant in Dunn's test, with the false discovery rate controlled using the Benjamini-Hochberg method. Ptr, *P. trichocarpa*; Egr, *E. grandis*; Lch, *L. chinense*. 109, SQK-PCS109; 111, SQK-PCS111. Bio, biological replicate.

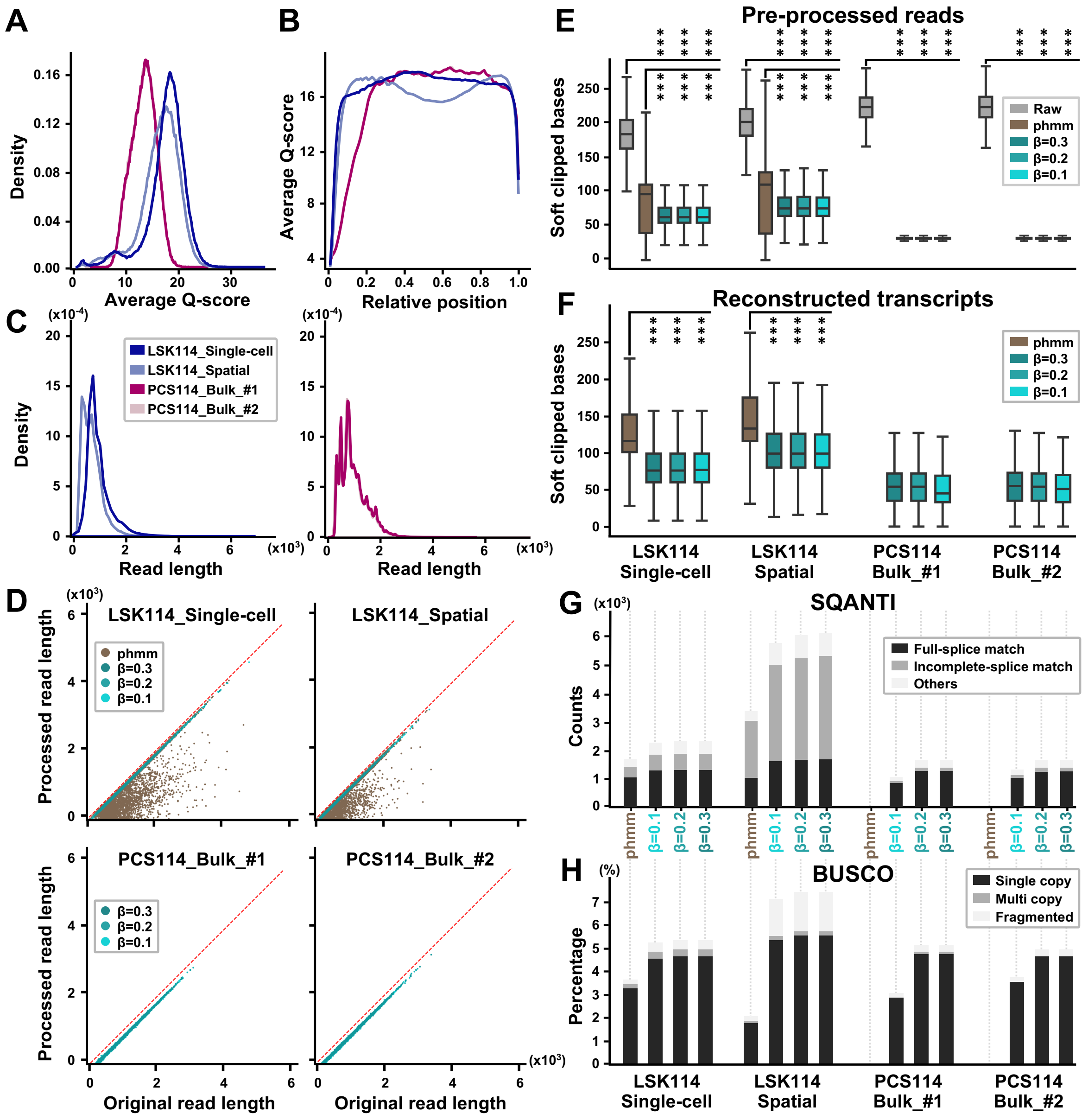

Figure S8. Comparison of NanoPrePro and Pychopper performance after the pre-processing of datasets generated by V14 kits. (A–C) Distribution of average Q-scores estimated by kernel density analysis **(A)**, average Q-scores across the relative position of reads **(B)**, and distribution of read lengths estimated by kernel density analysis **(C)** for each dataset, shown as dark blue for LSK114_Single-cell, light blue for LSK114_Spatial, deep magenta for PCS114_Bulk_#1, and pale rose for PCS114_Bulk_#2. **(D–H)** Scatter plots comparing read lengths before (x-axis) and after (y-axis) pre-processing **(D)**, number of soft-clipped bases in pre-processed reads with raw reads shown as control **(E)**, number of soft-clipped bases in reconstructed transcripts **(F)**, number of isoforms recovered in the reconstructed transcriptome classified by SQANTI structural categories, including full-splice match (all splice junctions match perfectly), incomplete-splice match (partial match to reference splice junctions), and others **(G)**, and percentage of BUSCO markers recovered in the reconstructed transcriptome categorized as single-copy, multi-copy, or fragmented BUSCOs **(H)**. Raw reads are shown in light grey. “phmm” (brown) denotes Pychopper run with the phmm backend. “β” (light, medium, and dark turquoise) denotes the β values used in NanoPrePro (0.1,

0.2, and 0.3, respectively). “***” indicated $p < 0.001$ in Dunn’s test, with the false discovery rate controlled using the Benjamini-Hochberg method. Single-cell, single-cell sequencing of the 293T cell line; Spatial, mouse brain spatial sequencing; Bulk, bulk RNA sequencing of mouse retina. #1 and #2 denote subsets 1 and 2.

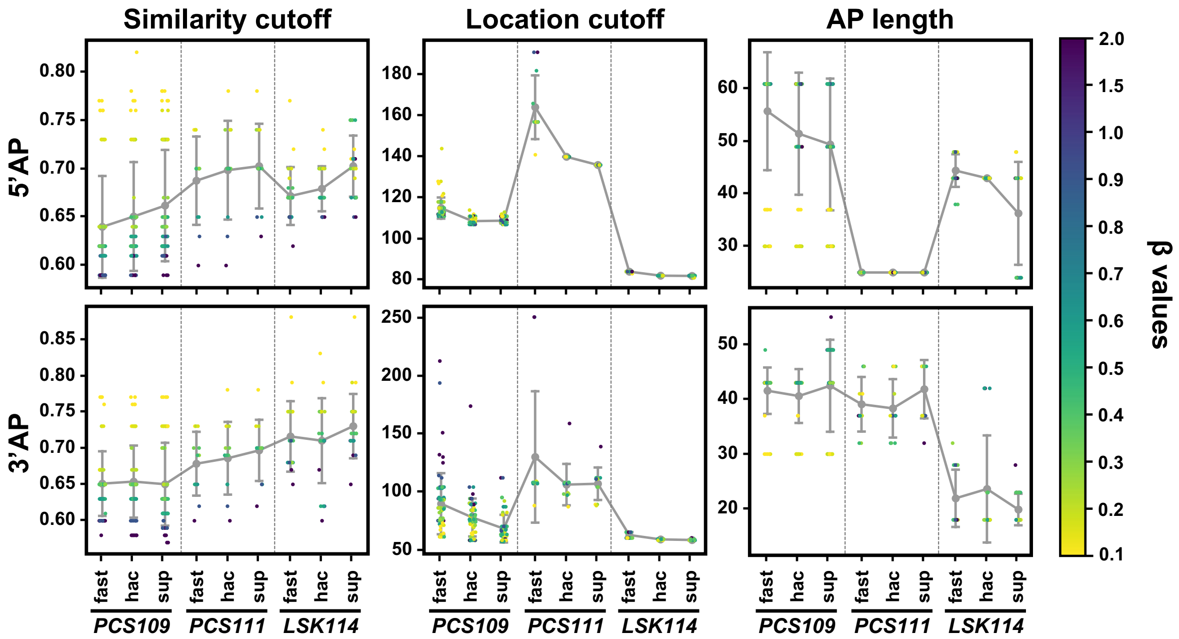

Figure S9. Optimized alignment cutoffs across sequencing kits, basecalling models, and β values. For each sequencing kit (PCS109, PCS111, and LSK114) and basecalling model (fast, hac, and sup), optimized cutoff values were determined under β values ranging from 0.1 (yellow) to 2.0 (purple). Colored (non-grey) points show the optimized cutoff values obtained under each β value, while grey points with error bars show the average value and its variation (mean ± standard deviation) across all β values for a given kit-model combination. The upper and lower panels show results for 5' and 3' adapters/primers (AP) alignments, respectively. The columns correspond to optimization for similarity cutoff (left), location cutoff (center), and AP length (right).

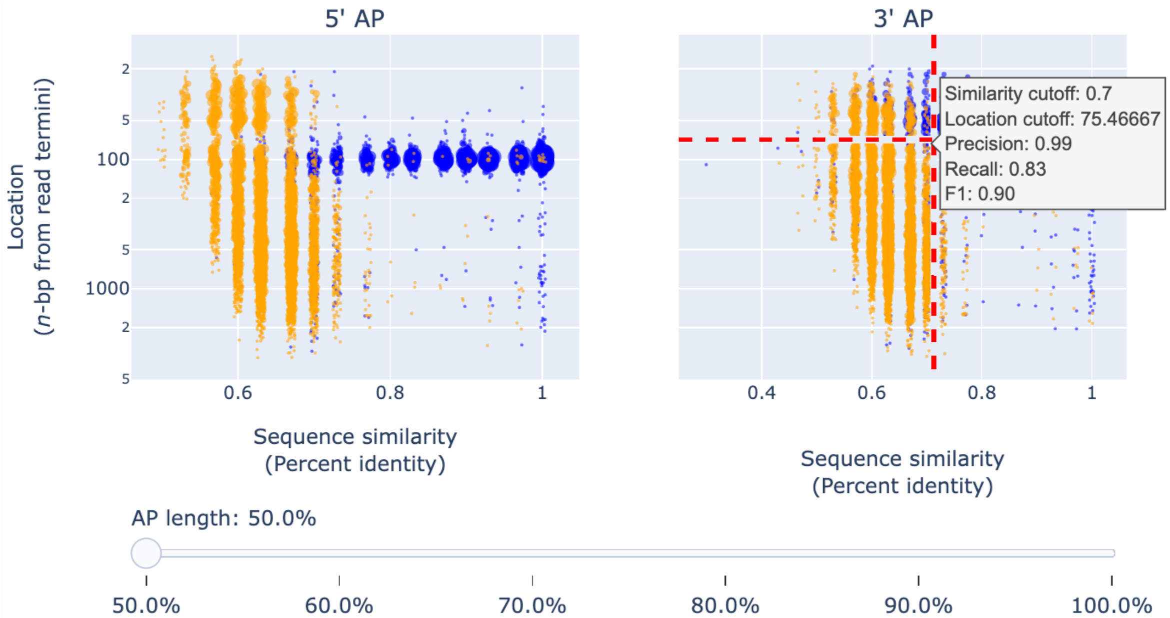

Fig. S10. Representative screenshot from an interactive HTML report generated by NanoPrePro that visualizes performance metrics under different alignment cutoffs. Simulated adapters/primers (AP) alignment results are shown as scatter plots for the 5' (left) and 3' (right) ends, with orange points indicating true alignments and blue points indicating random alignments. Each alignment is plotted by sequence similarity (x-axis) and alignment location relative to the read termini (y-axis). Red dashed lines indicate the cutoff values at the current cursor position, which update dynamically as the user moves the cursor. The hovering box displays the selected similarity and location cutoffs along with the corresponding precision, recall, and F1 score. A slider below the plots allows users to adjust the AP length cutoff and observe the resulting changes in performance metrics, where the percentage indicates the proportion of the full AP length (e.g., 50% represents half of the total AP length). This interactive visualization enables users to explore and compare performance metrics to facilitate the selection of optimal alignment cutoffs.

Table S1. The references and the corresponding statements for each pre-processing task.

Processing Step	Citations	Representative statement
Quality Filtering	Delahaye & Nicolas (2021)	The good news is that Nanopore quality scores are correlated with error rates, and this consistently for all studied species. This dependency is quasi-linear with a slight quadratic trend. Overall, the error rate E depends on the quality score Q following approximately equation E = 0.042Q2 − 2.68Q + 43.92 (R2 = 0.99) in the acceptable range Q 2 [7, 30].
	Soneson et al. (2019)	As we aligned unfiltered reads, the alignment rates were unsurprisingly only modest, varying across protocols between 62 and 69% for the genome alignment, and from 47 to 66% for the transcriptome alignment (Fig. 2a, Supplementary Fig. 4A). As expected, the unaligned reads were enriched for low base qualities (Supplementary Fig. 5A), and thus largely represented reads that would have been classified as failed during automatic filtering.
Full-length Identification	Soneson et al. (2019)	De novo as well as reference-based identification of transcripts suggested that a considerable number of the raw ONT reads are unlikely to represent full-length reference transcripts. This can have implications for transcript identification and quantification.
	Ibrahim et al. (2021)	However, as currently designed, ONT cannot perform true end-to-end sequencing as the 5 end of the RNA molecule is never fully sequenced due to the protein pore's inability to read the terminal 10-15 nucleotides (nt)
	Matsuo et al. (2021)	Benchmarking with MiSeq™ sequencing technology demonstrated the analytical advantage of sequencing the full-length 16S rRNA gene with MinION™, which could counteract the lower sequence accuracy and provide better resolution.
	Chen et al. (2020)	However, Illumina sequencing is generally insufficient for assembling complete genomic structures to investigate how genes are organized within genomes and whether these genes are located on the chromosome or plasmids of bacteria. This limitation is mainly attributed to the fact that short reads are not able to span the repetitive structures that extend beyond the maximum read length generated, thus producing unresolvable loops during genome assembly and resulting in an assembly consisting of many unordered contigs.

Processing Step	Citations	Representative statement
Strand Information	Sigurgeirsson et al. (2014)	Strand specific data gives more detailed and correct results than does non-stranded data as we show when estimating expression values and in assembling transcripts. Even well annotated genomes need improvements and corrections which can be achieved using strand specific data.
	Zhao et al. (2015)	Without strand information it is difficult and sometimes impossible to accurately quantify gene expression levels for genes with overlapping genomic loci that are transcribed from opposite strands.
	Katayama et al. (2005)	Transcription from the opposite (antisense) strand can produce transcripts that hybridize with the coding DNA strand, or with the antisense transcript, to interfere with transcription or mRNA stability.
	Barman et al. (2019)	An understanding of the regulatory mechanisms of antisense transcription, characterization of antisense transcripts and their modes of actions would be useful for the diagnosis, monitoring and targeted therapies of various diseases. Indeed, several antisense oligonucleotides are in clinical trials for the treatment of various diseases
Chimera Removal	White et al. (2017)	Although a non-negligible 1.7% of reads were found to have post-amplification chimeric elements, careful quality control of reads after long-read sequencing should be able to identify and exclude the majority of chimeric reads that are produced during a sequencing run.
	Tvedte et al. (2021)	Chimeric reads may hinder assembly-free analyses such as the validation of DNA integration into eukaryotic genomes via LGT. Although the overall frequency of chimeric reads is low, additional investigation of the occurrence and genome-wide distribution of chimeras is needed, particularly for eukaryotic genomes.
	Wick et al. (2018)	Reads will be binned if a sufficient match was found on either end, but if the two ends match different barcodes, the read will be considered a chimera and put in the 'none' bin.
	Heeger et al. (2018)	Chimeras were detected and removed with the uchime_denovo command in vsearch. Based on tests using mock community

Processing Step	Citations	Representative statement
		samples (see below), we determined this was a suitable method of chimera detection following the Read Processing stage (above). Only sequences that were classified as nonchimeric were used for further analysis.
	Laver et al. (2016)	p.R418X and p.L56 M occur 9 kb apart and, in this study, we used long-range PCR amplification and ONT and PacBio sequencing to phase these variants. From these analyses, we demonstrate PCR-chimera formation during PCR amplification and reference alignment bias are major pitfalls that need to be considered when attempting to phase variants using amplicon-based long-read sequencing technologies.
	Scheunert et al. (2020)	For optimal de novo assembly of Nanopore data, appropriate filtering of contaminants and chimeric sequences, as well as employing moderate read coverage, is essential.
Adapter Removal	Olsen et al. (2015)	For short-read data, the pipeline uses Trimmomatic for removing adapter contamination and low-quality regions. This has been shown to prevent the generation of adapter-chimeric contigs and to increase assembly contiguity.
	Sim et al. (2022)	We recommend a strict removal of an adapter containing read, versus an attempt at trimming out the adapter region and trying to retain a portion of the read as trimming could result in retention of chimeric molecules or other contaminating factors.
	Sturm et al. (2016)	Trimming of adapter sequences from short read data is a common preprocessing step during NGS data analysis. When performing paired-end sequencing, the overlap between forward and reverse read can be used to identify excess adapter sequences. This is exploited by several previously published adapter trimming tools. However, our evaluation on amplicon-based data shows that most of the current tools are not able to remove all adapter sequences and that adapter contamination may even lead to spurious variant calls.
	Chatterjee et al. (2012)	But our results strongly suggest that the trimming of adaptor sequences is an important step for improving mapping efficiency, supporting the conclusion of Gu et al.

Processing Step	Citations	Representative statement
	Ranjan et al. (2022)	Here, we introduce SNIKT, a command-line tool for sequence-independent visual confirmation and input-assisted removal of adapter contamination in whole-genome shotgun or metagenomic shotgun long-read sequencing DNA or RNA data.

Table S2. Functional comparison of goal-oriented tools for ONT read pre-processing.

Tasks	Guppy	Dorado	Prowler	Filtlong	NanoFilt	Porechop	Pychopper
Read quality filtering	O	O	O	O	O	-	O
Full-length read identification	-	-	-	-	-	-	O
Strand information preservation	-	O	-	-	-	-	O
Adapter/primer removal	O	O	-	-	O	O	O
Chimera identification	-	O	-	-	-	O	O
PolyA/T trimmer*	-	-	-	-	-	-	-

*Remaining an optional feature for read pre-processing based on previous studies.

Table S3. The quality and read-type proportions of 63 simulated ONT datasets.

Sample	Quality grade	Read-type proportion			Error rate
		Full-length	Truncated	Fusion	
Simulated dataset 1	low-quality	50%	50%	0%	13.88%
Simulated dataset 2	low-quality	50%	49%	1%	13.87%
Simulated dataset 3	low-quality	50%	45%	5%	13.86%
Simulated dataset 4	low-quality	50%	40%	10%	13.85%
Simulated dataset 5	low-quality	60%	40%	0%	13.86%
Simulated dataset 6	low-quality	60%	39%	1%	13.86%
Simulated dataset 7	low-quality	60%	35%	5%	13.85%
Simulated dataset 8	low-quality	60%	30%	10%	13.85%
Simulated dataset 9	low-quality	70%	30%	0%	13.86%
Simulated dataset 10	low-quality	70%	29%	1%	13.86%
Simulated dataset 11	low-quality	70%	25%	5%	13.84%
Simulated dataset 12	low-quality	70%	20%	10%	13.84%
Simulated dataset 13	low-quality	80%	20%	0%	13.85%
Simulated dataset 14	low-quality	80%	19%	1%	13.84%
Simulated dataset 15	low-quality	80%	15%	5%	13.84%
Simulated dataset 16	low-quality	80%	10%	10%	13.83%
Simulated dataset 17	low-quality	90%	10%	0%	13.83%
Simulated dataset 18	low-quality	90%	9%	1%	13.83%
Simulated dataset 19	low-quality	90%	5%	5%	13.83%
Simulated dataset 20	low-quality	90%	0%	10%	13.83%
Simulated dataset 21	low-quality	100%	0%	0%	13.83%
Simulated dataset 22	normal	50%	50%	0%	5.37%
Simulated dataset 23	normal	50%	49%	1%	5.36%
Simulated dataset 24	normal	50%	45%	5%	5.36%
Simulated dataset 25	normal	50%	40%	10%	5.36%
Simulated dataset 26	normal	60%	40%	0%	5.37%
Simulated dataset 27	normal	60%	39%	1%	5.37%
Simulated dataset 28	normal	60%	35%	5%	5.37%
Simulated dataset 29	normal	60%	30%	10%	5.37%
Simulated dataset 30	normal	70%	30%	0%	5.37%
Simulated dataset 31	normal	70%	29%	1%	5.37%
Simulated dataset 32	normal	70%	25%	5%	5.37%
Simulated dataset 33	normal	70%	20%	10%	5.36%
Simulated dataset 34	normal	80%	20%	0%	5.37%
Simulated dataset 35	normal	80%	19%	1%	5.36%
Simulated dataset 36	normal	80%	15%	5%	5.36%
Simulated dataset 37	normal	80%	10%	10%	5.36%

Table S3. The quality and read-type proportions of 63 simulated ONT datasets (Cont.).

Sample	Quality grade	Read-type proportion			Error rate
		Full-length	Truncated	Fusion	
Simulated dataset 38	normal	90%	10%	0%	5.37%
Simulated dataset 39	normal	90%	9%	1%	5.36%
Simulated dataset 40	normal	90%	5%	5%	5.37%
Simulated dataset 41	normal	90%	0%	10%	5.36%
Simulated dataset 42	normal	100%	0%	0%	5.37%
Simulated dataset 43	high-quality	50%	50%	0%	0.15%
Simulated dataset 44	high-quality	50%	49%	1%	0.15%
Simulated dataset 45	high-quality	50%	45%	5%	0.15%
Simulated dataset 46	high-quality	50%	40%	10%	0.15%
Simulated dataset 47	high-quality	60%	40%	0%	0.15%
Simulated dataset 48	high-quality	60%	39%	1%	0.15%
Simulated dataset 49	high-quality	60%	35%	5%	0.15%
Simulated dataset 50	high-quality	60%	30%	10%	0.15%
Simulated dataset 51	high-quality	70%	30%	0%	0.15%
Simulated dataset 52	high-quality	70%	29%	1%	0.15%
Simulated dataset 53	high-quality	70%	25%	5%	0.15%
Simulated dataset 54	high-quality	70%	20%	10%	0.15%
Simulated dataset 55	high-quality	80%	20%	0%	0.15%
Simulated dataset 56	high-quality	80%	19%	1%	0.15%
Simulated dataset 57	high-quality	80%	15%	5%	0.15%
Simulated dataset 58	high-quality	80%	10%	10%	0.15%
Simulated dataset 59	high-quality	90%	10%	0%	0.15%
Simulated dataset 60	high-quality	90%	9%	1%	0.15%
Simulated dataset 61	high-quality	90%	5%	5%	0.15%
Simulated dataset 62	high-quality	90%	0%	10%	0.15%
Simulated dataset 63	high-quality	100%	0%	0%	0.15%

Table S4. The throughput of the ONT SQK-PCS109 and SQK-PCS111 sequencing kits.

Sample	Library construction kit	Generated reads (M)	Estimated bases (GB)
Ptr_109_Bio1	SQK-PCS109	12.89	15.16
Ptr_109_Bio2		15.25	17.26
Egr_109_Bio1		24.24	16.78
Egr_109_Bio2		18.64	11.97
Lch_109_Bio1		15.30	14.69
Lch_109_Bio2		12.08	13.69
Ptr_111_Bio1	SQK-PCS111	31.71	22.90

Ptr, *P. trichocarpa*; Egr, *E. grandis*; Lch, *L. chinense*. 109, SQK-PCS109; 111, SQK-PCS111. Bio, biological replicate. M, million. GB, Gigabases.

Table S5. The error rates of the ONT transcriptomic datasets, estimated by mapping to the reference genome.

Sample	Error rate
Ptr_109_Bio1	7.13%
Ptr_109_Bio2	7.43%
Ptr_111_Bio1	8.62%
Egr_109_Bio1	8.75%
Egr_109_Bio2	7.81%
Lch_109_Bio1	6.66%
Lch_109_Bio2	7.38%
Cancer_109_#1	8.99%
Cancer_109_#2	9.84%

Ptr, *P. trichocarpa*; Egr, *E. grandis*; Lch, *L. chinense*. 109, SQK-PCS109; 111, SQK-PCS111. Bio, biological replicate.

Table S6. Average adapters/primers (AP) alignment cutoffs across eight PCS109 datasets* under varying β values.

β	5' AP			3' AP		
	AP length	Location cutoff	Similarity cutoff	AP length	Location cutoff	Similarity cutoff
0.1	36.9	116.4	0.77	37.8	56.8	0.76
0.2	40.4	116.6	0.72	39.3	56.8	0.72
0.3	52.5	116.6	0.67	46.4	56.8	0.68

*The eight PCS109 datasets included Ptr_109_Bio1, Ptr_109_Bio2, Egr_109_Bio1, Egr_109_Bio2, Lch_109_Bio1, Lch_109_Bio2, Cancer_109_#1, and Cancer_109_#2.

Table S7. The percentage of reads trimmed by more than 300 bases using Pychopper or NanoPrePro.

Sample	Pre-processor	% of reads trimmed more than 300 bases
Ptr_109_Bio1	Pychopper-phmm	2.4
	NanoPrePro-beta0.3	0
	NanoPrePro-beta0.2	0
	NanoPrePro-beta0.1	0
Ptr_109_Bio2	Pychopper-phmm	3.0
	NanoPrePro-beta0.3	0
	NanoPrePro-beta0.2	0
	NanoPrePro-beta0.1	0
Ptr_111_Bio1	Pychopper-phmm	0.5
	NanoPrePro-beta0.3	0.1
	NanoPrePro-beta0.2	0.1
	NanoPrePro-beta0.1	0.1
Egr_109_Bio1	Pychopper-phmm	1.9
	NanoPrePro-beta0.3	0
	NanoPrePro-beta0.2	0
	NanoPrePro-beta0.1	0
Egr_109_Bio2	Pychopper-phmm	2.0
	NanoPrePro-beta0.3	0
	NanoPrePro-beta0.2	0
	NanoPrePro-beta0.1	0
Lch_109_Bio1	Pychopper-phmm	1.1
	NanoPrePro-beta0.3	0
	NanoPrePro-beta0.2	0
	NanoPrePro-beta0.1	0
Lch_109_Bio2	Pychopper-phmm	1.3
	NanoPrePro-beta0.3	0
	NanoPrePro-beta0.2	0
	NanoPrePro-beta0.1	0
Cancer_109_#1	Pychopper-phmm	1.6
	NanoPrePro-beta0.3	0
	NanoPrePro-beta0.2	0
	NanoPrePro-beta0.1	0
Cancer_109_#2	Pychopper-phmm	1.6
	NanoPrePro-beta0.3	0
	NanoPrePro-beta0.2	0
	NanoPrePro-beta0.1	0

Ptr, *P. trichocarpa*; Egr, *E. grandis*; Lch, *L. chinense*. 109, SQK-PCS109; 111, SQK-PCS111. Bio, biological replicate.

Table S8. The assembly levels of reference genomes across different species domains.

Domain	Complete Genome	Chromosome	Scaffold	Contig	Total
Prokaryotes	11,276	1,694	38,977	35,450	87,397
Eukaryotes	151	1,951	6,719	2,516	11,337
Viruses	26,224	1,329	90	52	27,695

Reference

McClish, D.K. (1989). Analyzing a Portion of the ROC Curve. Medical Decision Making **9,** 190-195.
