# Supplementary material for: NanoPrePro: a fully equipped, fast, and memory-efficient preprocessor for nanopore transcriptomic sequencing
Source: Brief Bioinform. 2026 Feb 13;27(1):bbag063. doi: 10.1093/bib/bbag063 (PMC12903951; doi:10.1093/bib/bbag063)
Supplement: Supplementary_Methods_bbag063 [file supplementary_methods_bbag063.pdf]

Supplementary Methods

Algorithm for optimizing AP alignment cutoffs

As briefly outlined in the Results, we designed an algorithm to optimize alignment cutoffs for AP detection. The complete procedure is presented below in three parts:

(i) Simulation of true and random alignments (Fig. 3A and B). An AP sequence was aligned twice to each read based on edit distance: the first served as the true alignment, representing the best available placement of the AP. For the second alignment, the region identified in the first was masked. The AP sequence was then realigned, and this was treated as the random alignment. Each true/random alignment yields two features: sequence similarity (calculated as 1 minus the edit distance divided by the AP length) and aligned position on the reads (Fig. 3B).

(ii) F_β-score optimization (the red dashed lines in Fig. 3B). Using simulated data allowed an unambiguous distinction between true and random alignments. In real reads, however, such discrimination is more challenging, as some random alignments may display high sequence similarity or occur near the expected terminal regions of the read, making them appear indistinguishable from true alignments. To address this, simulated data were used to empirically determine optimal similarity and location cutoffs, thereby ensuring robust performance on real-world datasets. Upon the application of a similarity and location cutoff combination, namely the red dashed lines in Fig. 3B, we calculated the resulting precision and recall. True alignments located inside or outside the red dashed line are respectively considered as true positives and false negatives. On the other hand, random alignments located inside or outside the red dashed lines are respectively considered as false positives and true negatives. Precision was defined as the fraction of true positives among all positives (true positives + false positives) (Fig. 3B). Recall was defined

as the fraction of true positives among true alignments (true positives + false negatives) (Fig. 3B). Because we were accessing two metrics (precision and recall) simultaneously, F_β scores (the weighted harmonic mean of precision and recall) were used to evaluate the ideal cutoffs across a grid of similarity and location cutoff combinations.

(iii) Joint optimization of AP length, similarity, and location (Fig. 3C). The first two steps described above identify the combination of similarity and location cutoffs that yields the best performance for a fixed AP length. The procedure was then extended to "partial" APs, generated by clipping bases from the 5' end of 5' APs or from the 3' end of 3' APs to exclude low-quality regions at the sequence termini of real reads (Fig. S4B). For each partial AP, the best combination of similarity and location cutoff was identified as described in the first two steps. The joint combination of AP length, similarity cutoff, and location cutoff that achieves the highest F_β score was selected as the parameter for AP alignment.

Plant materials and xylem tissue collection

P. trichocarpa (clone Nisqually-1) and *E. grandis* trees were grown using peat moss:vermiculite (3:1) and maintained under a 16-hour photoperiod at 25 °C in a greenhouse. The xylem tissues of *P. trichocarpa* and *E. grandis* were harvested from 9-month-old plants with heights of 2.7 m and 1.8 m, respectively. The xylem tissues were collected following the same procedure described in our previous works [1-3]. In brief, the stems were chopped down, the leaves were removed, and the bark was peeled off. The xylem tissues exposed on the debarked stem surface were scraped off using single-edged blades. The collected xylem tissues were frozen using liquid nitrogen and stored at -80°C. *L. chinense* trees were planted at a field site at the Highland Experimental Farm of National Taiwan University. The xylem tissues from *L. chinense* were collected following the same procedure described above.

RNA isolation

Xylem total RNA was extracted using the CTAB method. The xylem tissues were powdered with liquid nitrogen, mixed with pre-warmed CTAB extraction buffer (2% CTAB; 0.1 M Tris-HCl, pH 9.0; 25 mM EDTA, pH 8.0; 2 M NaCl; 1% PVP-40; 2% β-mercaptoethanol; 50 mM ascorbic acid; pre-warmed at 65°C for 20 minutes), incubated at 65°C for 10 minutes, and then centrifuged at 12,000 g for 5 minutes at room temperature. The supernatant was mixed with an equal volume of chloroform:isoamyl alcohol (24:1 v/v) and centrifuged at 12,000 g for 10 minutes at room temperature. This chloroform:isoamyl alcohol extraction step was repeated once. The aqueous phase was collected, mixed with 1/3 volume of 8 M LiCl, and kept at 4°C for 14 hours. The total RNA was pelleted by centrifugation at 12,000 g for 20 minutes at 4°C, dissolved in the RLT buffer, and purified using the RNeasy Plant Mini Kit (Qiagen, Valencia, CA, USA) according to the manufacturer's instructions. Only high-quality RNA samples (RIN ≥ 8.0, as assessed using the Bioanalyzer 2100 from Agilent Technologies, Santa Clara, CA) were used for ONT sequencing.

ONT sequencing and read sampling

Sequencing libraries were prepared from xylem total RNA using the Nanopore cDNA-PCR sequencing kits (SQK-PCS109 and SQK-PCS111, Oxford Nanopore Technologies) according to the manufacturer's instructions. For library construction, total RNA samples were reverse transcribed using oligo(dT) adapters to capture mRNA, followed by PCR amplification and ligation with ONT sequencing adapters. The constructed libraries were loaded onto FLO-MIN106 (R9) flow cells installed on a MinION sequencing device and sequenced for a maximum duration of 72 hours. A hundred thousand sequence records were randomly sampled from each run using the "random" package from Python 3.10 with a fixed seed (42). Basecalling was performed using

Dorado (version 0.9.5, the latest version of Dorado supporting data generated by R9 flow cells) with the basecalling model “dna_r9.4.1_e8_sup@v3.6” and with the parameter “--no-trim” to disable adapter trimming (https://github.com/nanoporetech/dorado). All basecalled reads were used in following analyses regardless of their average Q-score.

Publicly available LSK114 and PCS114 datasets

For SQK-LSK114 sequencing data, two datasets were obtained from the Oxford Nanopore Open Data Project (accessed on August 26 from https://registry.opendata.aws/ont-open-data): spatial transcriptomic sequencing of the mouse brain and single-cell sequencing of the human 293T cell line. From each dataset, one raw signal file (POD5) was sampled and basecalled with Dorado (version 1.1.1) using the model “dna_r10.4.1_e8.2_400bps_sup@v5.2.0”. For SQK-PCS114 sequencing data, two subsets (10,000 reads each) were sampled from a published bulk RNA sequencing dataset of mouse retina [4]. The dataset was provided in basecalled FASTQ format. Sequencing was performed on an R10.4 flow cell, and basecalling was carried out using the model “dna_r10.4.1_e8.2_400bps_sup@v4.3.0”.

ONT data simulation

Simulated ONT datasets were generated using Badread (version 0.4.0) [5]. Random fragments were first sampled from the *Populus trichocarpa* reference genome (assembly version 4 from Phytozome [6]) using a fixed random seed (42). The length distribution of the sampled fragments followed a mean of 3000 bases and a standard deviation of 1000 bases.

According to the structure of ONT libraries (Fig. S1), adapters/primers would be attached to the sampled fragments. The simulated sequences of the 5’ and 3’ primers were based on the SQK-PCS109 kit. The simulated adapters were self-defined sequences, as ONT did not disclose

the “sequencing adapter” sequence. The simulated sequences of the 5’ and 3’ adapters/primers were shown as follows:

The 5’ adapter/primer sequences:

ATCGATCGATCGATCGATCGATCGATCGATCGATCGATCGATCGATCGATCGATCGATCGA
TCGGGGGATCGCCTACCGTGACAAGAAAGTTGTCGGTGTCTTTGTGTTTCTGTTGGT
GCTGATATTGC

The 3’ adapter/primer sequences:

GAAGATAGAGCGACAGGCAAGTCACAAAGACACCGACAACTTTCTTGTCACGGTAG
GCGATGGGGATCGATCGATCGATCGATCGATCGATCGATCGATCGATCGATCG

A portion of the 5’ and 3’ simulated adapters/primers, with an average length of 61% of the complete adapter/primer length, was then attached to the reads to mimic reduced sequence quality at the read terminals.

Three types of reads were simulated: full-length reads were created by attaching both 5’ and 3’ adapters/primers; truncated reads were simulated by attaching only the 5’ adapter/primer; and chimeric reads were generated by joining two full-length reads. Three sets of parameters were used to generate reads of varying quality. For high-quality reads, the parameters were “--identity 30,3 --error_model random --qscore_model ideal --glitches 0,0,0”. For normal (median-quality) reads, the parameters were “--identity 95,99,2.5 --error_model nanopore2023 --qscore_model nanopore2023 --glitches 10000,25,25”. For low-quality reads, the parameters were “--identity 95,99,2.5 --error_model nanopore2023 --qscore_model nanopore2023 --glitches 1000,100,100”. The full-length, truncated, and chimeric reads of varying qualities were ultimately sampled using

the “random” package from Python 3.10 with a fixed seed (42) and pooled into 63 datasets (Table S2).

The parameters used for performance comparison between Pychopper and NanoPrePro

A single thread was assigned to Pychopper (version 2.7.10) and NanoPrePro, using the parameters “-t 1” for Pychopper and “--process 1” for NanoPrePro. The number of reads sampled during optimization/autotuning was set to 100,000 using the parameter “-Y 100000” for Pychopper and “-n 100000” for NanoPrePro. Adapter/primer sequences for alignments were set for Pychopper using the “--kit” parameter with the name of the corresponding library construction kit. For NanoPrePro, primer sequences provided by ONT were used for the SQK-PCS109, SQK-PCS111, and SQK-PCS114 kits. For SQK-LSK114, the official primer sequences were further extended with custom oligos described in the relevant online protocols (document versions STS_9223_v114_revA_09Jun2025 and SST_9198_v114_revO_09Jun2025). Adapter trimming and read orientation were performed by default in Pychopper, while these functions were executed in NanoPrePro using the parameters “--trim_adapter --orientation 1”. PolyA/T trimming was a feature exclusive to NanoPrePro and was activated by specifying “--trim_poly --poly_w 6 --poly_k 4” and “A{100}”. These settings enabled NanoPrePro to identify and remove polyA/T sequences with a maximum length of 100 bases. Length filtering was not enforced on the output reads, with Pychopper using “-z 0” and NanoPrePro using “--filter_short 0”. The quality filtering threshold was set to an average Q-score of 7 using “-Q 7” for Pychopper and “--filter_lowq 7” for NanoPrePro. The output files were managed differently in Pychopper and NanoPrePro. In Pychopper, the output file for full-length reads was specified as the last positional argument, and the output files for truncated and chimeric reads were designated using the “-u” and “-w” parameters, respectively. In NanoPrePro, the output files for full-length, truncated, and chimeric

reads were specified using the parameters "--output_full_length", "--output_truncated", and "--output_fusion", respectively.

For the comparison of performance between NanoPrePro and Pychopper, Pychopper was executed with different alignment backends using "-m phmm" and "-m edlib". NanoPrePro was run with varying degrees of stringency by adjusting the "--beta" parameter.

Performance metrics: precision, recall, and F_β score

Precision, recall, and the F_β score were used to evaluate the performance of Pychopper and NanoPrePro. Precision measures the percentage of true positives among all cases classified as positive. It represented the percentage of actual true alignments among all alignments classified as true, or the percentage of actual full-length reads among all reads classified as full-length in this study.

$$\text{Precision} = \frac{\text{True positive}}{\text{True positive} + \text{False positive}}$$

Recall measures the ratio of identified true positives to all actual positive events in a dataset. In this study, it was defined as the ratio of identified true alignments to all true alignments, or as the ratio of identified full-length reads to all full-length reads in a dataset.

$$\text{Recall} = \frac{\text{True positive}}{\text{True positive} + \text{False negative}}$$

The F_β score is the weighted harmonic mean of precision and recall, where β is a factor that determines the relative weight of precision and recall in the combined score.

$$F_\beta = \frac{1 + \beta^2}{\frac{1}{precision} + \frac{\beta^2}{\text{recall}}}$$

Reference-free transcript reconstruction

Reference-free transcript reconstruction was performed using RATTLE [7] with full-length reads identified and pre-processed by Pychopper and NanoPrePro. The "rattle cluster" sub-module of RATTLE was employed with parameters "--iso --rna" to perform isoform-level clustering and to prevent reverse-complementation of the reads, given that all the reads were in the sense-strand direction after pre-processing by Pychopper and NanoPrePro. Error correction was applied to read clusters using the sub-module "rattle correct" with default parameters. The corrected reads were then refined with the sub-module "rattle polish" using the "--rna" parameter.

Genomic and transcriptomic alignment

The reference genome and transcriptome for *Populus trichocarpa* (version 4.1) and *Eucalyptus grandis* (version 2.0) were downloaded from Phytozome [6]. The first release of the *Liriodendron chinense* reference genome and annotation was downloaded from the TreeGenes Project (accessed on Oct 2, 2024) [8]. Genomes and annotations (release 114) for human (GRCh38.p14) and mouse (GRCm39) were obtained from Ensembl [9]. Alignments to the reference genome were performed using minimap2 (version 2.30) [10] with the parameters "-ax splice --secondary=no" to accommodate long deletions caused by introns and report only primary alignments.

BUSCO completeness and structural categorization of reconstructed transcripts

BUSCO (version 6.0.0) [11] was used to evaluate the completeness of reconstructed transcripts with the parameter "-m transcriptome." Lineage datasets were selected according to species: "malpighiales_odb12" for *P. trichocarpa*, "eudicotyledons_odb12" for *E. grandis*, "embryophyte_odb12" for *L. chinense*, "primates_odb12" for human, and "rodentia_odb12" for

mouse. Transcript structures were further characterized using SQANTI (version 5.0.0) [12], which assigned the reconstructed transcripts to known isoforms and categorized them into structural classes: full-splice match (all splice junctions match perfectly), incomplete-splice match (partial match to reference splice junctions), and others. Transcript coverage was estimated by measuring the distance between each reconstructed transcript and the transcription start and termination sites of its assigned isoform.

References

1. Lin Y-C, Li W, Chen H et al. A simple improved-throughput xylem protoplast system for studying wood formation, Nat Protoc 2014;**9**:2194-2205.
2. Lin YC, Li W, Sun YH et al. SND1 transcription factor-directed quantitative functional hierarchical genetic regulatory network in wood formation in Populus trichocarpa, Plant Cell 2013;**25**:4324-4341.
3. Yeh CS, Wang Z, Miao F et al. A novel synthetic-genetic-array-based yeast one-hybrid system for high discovery rate and short processing time, Genome Res 2019;**29**:1343-1351.
4. Wang M, Li Y, Wang J et al. Integrating short-read and long-read single-cell RNA sequencing for comprehensive transcriptome profiling in mouse retina, Genome Res 2025;**35**:740-754.
5. Wick RR. Badread: simulation of error-prone long reads, J Open Source Softw 2019;**4**:1316.
6. Goodstein DM, Shu S, Howson R et al. Phytozome: a comparative platform for green plant genomics, Nucleic Acids Res 2012;**40**:D1178-1186.
7. de la Rubia I, Srivastava A, Xue W et al. RATTLE: reference-free reconstruction and quantification of transcriptomes from Nanopore sequencing, Genome Biol 2022;**23**:153.
8. Falk T, Herndon N, Grau E et al. Growing and cultivating the forest genomics database, TreeGenes, Database 2019;**2019**.
9. Harrison PW, Amode MR, Austine-Orimoloye O et al. Ensembl 2024, Nucleic Acids Res 2023;**52**:D891-D899.
10. Li H. Minimap2: pairwise alignment for nucleotide sequences, Bioinformatics 2018;**34**:3094-3100.
11. Tegenfeldt F, Kuznetsov D, Manni M et al. OrthoDB and BUSCO update: annotation of orthologs with wider sampling of genomes, Nucleic Acids Res 2024;**53**:D516-D522.
12. Pardo-Palacios FJ, Arzalluz-Luque A, Kondratova L et al. SQANTI3: curation of long-read transcriptomes for accurate identification of known and novel isoforms, Nat Methods 2024;**21**:793-797.
